# Disruption of the *protein kinase N* gene of *Drosophila melanogaster* Results in the Recessive *delorean* Allele (*pkn^dln^*) With a Negative Impact on Wing Morphogenesis

**DOI:** 10.1534/g3.114.010579

**Published:** 2014-02-13

**Authors:** Georgette L. Sass, Bruce D. Ostrow

**Affiliations:** Department of Biology, Grand Valley State University, Allendale, Michigan 49401

**Keywords:** PKN, wing morphology, Rho effector, signal transduction, JNK pathway

## Abstract

We describe the *delorean* mutation of the *Drosophila melanogaster* *protein kinase N* gene (*pkn^dln^*) with defects in wing morphology. Flies homozygous for the recessive *pkn^dln^* allele have a composite wing phenotype that exhibits changes in relative position and shape of the wing blade as well as loss of specific vein and bristle structures. The *pkn^dln^* allele is the result of a *P*-element insertion in the first intron of the *pkn* locus, and the *delorean* wing phenotype is contingent upon the interaction of insertion-bearing alleles in *trans*. The presence of the insertion results in production of a novel transcript that initiates from within the 3′ end of the *P*-element. The *delorean*-specific transcript is predicted to produce a wild-type PKN protein. The *delorean* phenotype is not the result of a reduction in *pkn* expression, as it could not be recreated using a variety of wing-specific drivers of *pkn*-RNAi expression. Rather, it is the presence of the *delorean*-specific transcript that correlates with the mutant phenotype. We consider the *delorean* wing phenotype to be due to a pairing-dependent, recessive mutation that behaves as a dosage-sensitive, gain of function. Our analysis of genetic interactions with *basket* and *nemo* reflects an involvement of *pkn* and Jun-terminal kinase signaling in common processes during wing differentiation and places PKN as a potential effector of Rho1’s involvement in the Jun-terminal kinase pathway. The *delorean* phenotype, with its associated defects in wing morphology, provides evidence of a role for PKN in adult morphogenetic processes.

A cell’s ability to receive information and respond appropriately requires a process of signal transduction, whereby an external stimulus is coupled to transducers that relay information in from the cell surface. Signaling pathways often connect stimuli with specific protein modifications that ultimately alter protein function to mediate the desired response. Any given cell type uses many pathways, often with cross talk between them. Despite such complexity, components of signal transduction pathways have been highly conserved (Pires-daSilva and Sommer 2006).

Rho family GTPases are one such conserved family of proteins, which belong to the Ras-like GTPase superfamily (Wennerberg *et al.* 2005; Boureux *et al.* 2007), acting as signal transducers that regulate and integrate a wide range of cellular processes, including actin reorganization, cell adhesion, cell polarity, cell-cycle progression, and apoptosis (Coleman and Olson 2002; Bustelo *et al.* 2007; Guilluy *et al.* 2011; Spiering and Hodgson 2011; Ridley 2012). Rho GTPases are monomeric guanosine-5’-triphosphate (GTP)-binding proteins (G proteins) that act as molecular switches, cycling between a GDP-bound inactive state and GTP-bound active state in response to external stimuli. In their active GTP-bound configuration, Rho GTPases bind to and activate downstream effectors. More than 60 effector proteins have been identified based on their interaction with the three best-studied members of the Rho GTPase family: Rho, Rac, and Cdc42 (Bishop and Hall 2000; Iden and Collard 2008; Hall 2012). The considerable interest in understanding Rho GTPase family members and their effectors is warranted, given their pivotal role in cellular processes, including establishing and maintaining cell polarity and cell adhesion, and because altered activity of Rho GTPases helps drive malignant transformations (Iden and Collard 2008; Vega and Ridley 2008; Karlsson *et al.* 2009; Provenzano and Keely 2011; Unsal-Kacmaz *et al.* 2012).

Protein kinase N (PKN) is a downstream effector of both Rac1 and Rho1 that interacts directly with these GTPases in their active, GTP-bound state (Vincent and Settleman 1997; Lu and Settleman 1999). Activation by either Rac1 or Rho1 occurs via association with distinct regulatory sequences found at the N-terminus of PKN, known as HR1 repeats. HR1 repeats exhibit similarity to the leucine zipper structural motif and adopt an antiparallel coiled-coil structure (Maesaki *et al.* 1999). Within its kinase domain, PKN is highly similar to protein kinase C (Mukai and Ono 1994) and, as such, is a member of the larger AGC kinase subfamily of serine-threonine protein kinases (Pearce *et al.* 2010). PKN homologs are found in invertebrates and vertebrates (Palmer *et al.* 1995; Kitagawa *et al.* 1995; Mukai *et al.* 1995; Ueno *et al.* 1997; Stapleton *et al.* 1998). In mammals, PKN has demonstrated involvement in the regulation of cytoskeletal reorganization (Mukai *et al.* 1997; Vincent and Settleman 1997), cell adhesion (Calautti *et al.* 2002), cell-cycle regulation (Mukai 2003), and tumorigenesis (Metzger *et al.* 2003; Leenders *et al.* 2004). There are three mammalian paralogs of PKN (Mukai 2003), presented in the literature under various names (PKN1, PKN2, PKN3, PKNα, PKNβ, PKNγ, PRK1, PRK2). PKN1 and PKN2 are expressed widely, whereas the expression of PKN3 is more restricted (Mukai and Ono 2006; Lachmann *et al.* 2011) and studied more often in cultured cells (Mukai *et al.* 1996). Although there is a great deal of information about PKN function from cultured cells, few whole-animal studies have been conducted. Furthermore, because the three types of mammalian PKN exhibit overlapping patterns of expression, it is sometimes difficult to know which paralog is functionally relevant or whether functional redundancy is involved. Therefore, we sought a simpler system in which to study PKN function.

The fruit fly *Drosophila melanogaster* offers a superb system to study the normal developmental role of Rho family effectors (Johndrow *et al.* 2004). The protein structure of the single *Drosophila* PKN is very similar to its well-characterized mammalian orthologs, containing an N-terminal region with three HR1 repeats, followed by a C2 domain related to the calcium-dependent membrane-targeting domain of protein kinase C and finally a C-terminal kinase domain (Ueno *et al.* 1997). In *Drosophila*, *protein kinase N* (*pkn*) has been implicated in the process of dorsal closure during embryogenesis, involving the migration and fusion of epidermal cells that resembles wound healing (Lu and Settleman 1999). Loss of PKN results in dorsal closure failure in ~10% of homozygous *pkn^06736^* individuals (Lu and Settleman 1999). Regulation and rearrangement of the cytoskeleton is required as cells change shape and alter contacts with other cells during dorsal closure. It is known that Rho GTPase-dependent signaling pathways participate in dorsal closure, because both Rac1 and Rho1 loss-of-function mutants exhibit defects in the processes required for dorsal closure, including actin reorganization, cell movement, and cell-cell contact (Harden *et al.* 1999; Baek *et al.* 2010; Bahri *et al.* 2010). However, the exact mechanism by which PKN acts in its capacity as a Rac/Rho target effector in fly development has yet to be determined.

The loss-of-function, dorsal closure phenotype associated with *pkn^06736^* is relatively mild (~10% of embryos with the phenotype) and may reflect functional redundancy with components of the Jun-terminal kinase (JNK) pathway, which is also required for dorsal closure in *Drosophila*. Previous work has suggested that in its capacity as a Rho1 effector, PKN is involved in a JNK-independent instructive signal for dorsal closure (Lu and Settleman 1999). PKN function is required for viability in *Drosophila*, as evidenced by a multiphasic lethal phenotype that fails to generate homozygous *pkn^06736^* adults (Lu and Settleman 1999). However, this does not provide the insight needed to determine the nature of PKN’s effector function. For this reason, we sought to analyze additional mutant alleles of the *pkn* gene to better understand the function of the PKN protein. Here we describe a new allele of *Drosophila pkn* we called *delorean* (*pkn^dln^*), which was identified from the Kiss *P*-element mutagenesis stock collection of *P[lacW]* transposon insertions on the second chromosome (Torok *et al.* 1993). The *delorean* phenotype, with its associated defects in wing morphology, provides evidence for a novel role of PKN in morphogenetic processes and provides new insights into the effector function of PKN.

## Materials and Methods

### Fly stocks and genetics

Fly stocks were maintained at 25° on hydrated fly food (Carolina Biological Supply) supplemented with live yeast. Flies were anesthetized using carbon dioxide. Where heteroallelic interactions were studied for phenotypic analysis, matings between strains were carried out at 25° using reciprocal crosses.

The stock *y^1^w^67c23^*; *P[w^+mC^ = lacW]l(2)k06808/CyO* (referred to as *P[lacW]k06808*) was obtained from the Kiss *P*-element mutagenesis stock collection (Torok *et al.* 1993). The *basket (bsk^1^*) *pkn^dln^* chromosome was generated in our lab by recombining *bsk^1^* (using Bloomington stock #3088) and *pkn^dln^* alleles. All other stocks used in this study were obtained from Bloomington and Umea stock centers or created in our lab. The *P[lacW]k06808* insertion chromosome was initially outcrossed for eight generations to a Bloomington stock isogenic for chromosome 2 (*y^1^w^67c23^*; *iso-2)* and rebalanced over *y^+^CyO* (kindly provided by Karen Blochlinger). Subsequently, the *P[lacW]k06808* insertion chromosome was outcrossed for >40 generations to a *w^1118^* strain (*w^1118^*; *iso-2*) and maintained by selection for the *mini-white* marker of *P[lacW]*.

### Transposon excision

*P[lacW]k06808/CyO* individuals were crossed to w*; *Sp/CyO*; *Δ2-3 Dr/TM6*. Dysgenic *P[lacW]k06808/CyO*; *Δ2-3 Dr/+* offspring were mated to *y^1^w^1^/FM6*; *Pin^1^/CyO* flies. Individual white-eyed *y^1^w^1^/Y*; *ΔP/CyO* males were backcrossed to *P[lacW]k06808/CyO* virgins to test for genetic interaction and to establish individual balanced revertant stocks.

### Wing morphology analysis

Wings were dissected dry, transferred to glass microscope slide with isopropanol and mounted in AquaPolymount (Polysciences, Inc.). Anterior wing margin bristles and sensillae were viewed under brightfield optics using a Nikon E200 microscope at 400× magnification. For each wing, we counted the number of twin campaniform sensillae, stout bristles, dorsal recurved bristles, ventral recurved bristles, ventral slender bristles, and domed sensillae on longitudinal vein L3 and on the anterior crossvein. Sample size for each genotype varied from 5 to 32. Median values were subjected to a Kruskal-Wallis test. For those traits with a *P*-value < 0.001, a Mann-Whitney *U* test was performed on paired combinations of all genotypes.

### Determination of *delorean* homozygote viability

Virgins of the genotype *pkn^dln^*/*CyO*, *P{w^[+mC]^Act5C-GFP}JMR1 pkn^+^* were mated to *pkn^dln^*/*pkn^dln^* males and then allowed to lay eggs on a collection plate (3% agar, 20% apple juice, 10% sucrose) for 4−6 hr at 25°. Adults were removed and embryos were allowed to develop. First instar larvae were sorted for the presence or absence of the green fluorescent protein (GFP) marker associated with the *CyO* balancer, placed on Carolina food supplemented with yeast at 25° and observed throughout development to adulthood. We would expect that if *pkn^dln^*/*pkn^dln^* homozygotes are 100% viable they should represent half of the progeny from these matings.

### Polytene chromosome *in situ* hybridization

*In situ* hybridization was carried out essentially according to Pardue (1986) with the following exceptions: Salivary glands were dissected from wandering third instar larvae in 50% acetic acid and squashed in 45% acetic acid, 18% lactic acid on glass microscope slides. Hybridization was performed overnight at 55° in hybridization solution (0.6 M NaCl; 50 mM NaPO_4_, pH 7.2; 500 μg/mL salmon sperm DNA; 5× Denhardt’s solution) containing 3 μg/mL digoxigenin-labeled *Xba*I-linearized *P[lacW]* probe (kindly provided by Ed Giniger). Bound probe was detected using an alkaline phosphatase-based detection kit (Boehringer Mannheim). Chromosomes were counterstained with Giemsa (Fluka) and viewed in phase contrast through a Nikon Axiophot microscope.

### Genomic analysis of transposon insertion alleles and derivative alleles

Genomic DNA (gDNA) was prepared from 50 homozygous *P[lacW]k06808* adults according to Ashburner (1989). Frozen flies were homogenized in buffer (0.1 M Tris-HCl, pH 9; 0.1 M ethylenediaminetetraacetic acid; 1% sodium dodecyl sulfate; 0.5% diethylpyrocarbonate). The homogenate was incubated at 63° for 30 min, clarified with 1 M potassium acetate, and precipitated with isopropanol. Then, 1 μg of gDNA was digested individually with restriction endonucleases *Bam*HI or *Eco*RI (New England Biolabs) and phenol/chloroform extracted and ethanol precipitated. Digested gDNA preparations were individually diluted to 1 μg/mL in TE (0.01 M Tris-HCl, pH 8.0; 0.001 M ethylenediaminetetraacetic acid), self-ligated with T4 ligase (Promega), and transfected into XL-1 Blue competent *E. coli* cells (Stratagene). Transformants were plated on LB containing 65 μg/mL ampicillin. Two positive colonies of each type were individually grown in LB-Amp broth. Plasmid DNA was prepared by alkaline lysis, purified through a QIAGEN-tip 100 column (QIAGEN), and sequenced with primers near the ends of *P[lacW]*. Primer sequences were *Eco*Right1: 5′CGTTAAGTGGATGTCTCTTGC3′, BamLeft1: 5′TTCCTCTCAACAAGCAAAC3′ and BamRight1: 5′GCGTCGATTTTTGTGATGCTC3′. DNA sequencing was performed by the Iowa State University DNA Sequencing Facility.

The insertion sites of the transposons associated with the *P[lacW]l(2)k11209* and *P[PZ]l(2)rG232^rG232^* strains were determined using the standard inverse polymerase chain reaction (PCR) protocol available from the Berkeley Drosophila Genome Project (http://www.fruitfly.org/about/methods/inverse.pcr.html) with the following specifications. The restriction endonuclease *Hin*P1I (New England Biolabs) was used to digest gDNA with the P5′ region of the transposon amplified using primers Plac4 5′ACTGTGCGTTAGGTCCTGTTCATTGTT3′ and Plac1 5′CACCCAAGGCTCTGCTCCCACAAT3′. The P3′ region of the transposon was amplified using primers Pry1 5′CCTTAGCATGTCCGTGGGGTTTGAAT3′ and Pry2 5′CTTGCCGACGGGACCACCTTATGTTATT3′. Sequence was obtained from the amplified P5′ product using Splac2 5′GAATTCACTGGCCGTCGTTTTACAA3′ or from the amplified P3′ product using one of the original PCR primers. DNA was sequenced on an Applied Biosystems 3130*xl* Genetic Analyzer at the Annis Water Resources Institute Sequencing Facility of Grand Valley State University.

Analysis of imprecise excision derivatives used gDNA prepared from homozygous adults as described previously. Then, 5 μL of gDNA was amplified using LongAmp Taq polymerase (New England Biolabs) with 0.4 μM oligonucleotide primers in an Eppendorf Mastercycler. PCR conditions were as follows: initial denaturation at 98° for 5 min followed by 35 cycles of 98 for 30 sec, 55° for 40 sec, 65° for 9 min. Primers used were plac4 5′ACTGTGCGTTAGGTCCTGTTCATTGTT3′, placWFor1 5′CACGCGGACTATTCTGCAAC3′, Ex1For2 5′GGAAGCAGTCGGCGTTATG3′, Ex2Rev2 5′GATGCCGTAGCTTCTTGTTG3′, and plac7 5′CGTGGTGTCACGCTCGTCGTT3′. PCR products were size selected in an agarose gel, purified using MinElute columns (QIAGEN), and sequenced as described previously.

### Rapid amplification of 5′ cDNA ends (5′ RACE)

The *pkn^dln^* and *Df(2R)w45-30n* chromosomes were rebalanced separately over *CyO*, *P{w^[+mC]^ActGFP}JMR1*, and then the lines were mated together. Third instar larvae were sorted for the presence or absence of the GFP marker associated with the *CyO* balancer, placed on Carolina food supplemented with yeast at 25°, and allowed to pupate. One-day-old pupae were homogenized in buffer RLT (QIAGEN), lysates were passed through QiaShredder columns (QIAGEN), and total pupal RNA was purified over RNeasy columns (QIAGEN). RNA samples were treated with DNAse (New England Biolabs) and repurified over RNeasy columns. Then, 7 μL of each RNA sample was subjected to RNA ligase-mediated rapid amplification of 5′ cDNA ends (RLM-RACE) according to manufacturer’s protocol (Invitrogen GeneRacer kit). RNAs were reverse transcribed with a primer that binds within exon 7 of *pkn* (5′-TCCGGACTGCAGTGACGTGTAGGTG-3′) then amplified with the GeneRacer 5′ primer (5′-CGACTGGAGCACGAGGACACTGA-3′) and a primer that binds within exon 5 of *pkn* (5′-AATGGCCTCCTTGATCTCCT-3′). PCR products were cloned into pCR4-TOPO (Invitrogen) and transfected into One Shot TOP10 *E. coli* cells (Invitrogen) with selection on carbenicillin (0.075 mg/mL). Recombinant plasmid DNA was sequenced as described previously. Sequence data from this article have been deposited with NCBI GenBank under accession no. KJ201875.

## Results

### *delorean* mutants have defects in wing development

**Figure 1:**
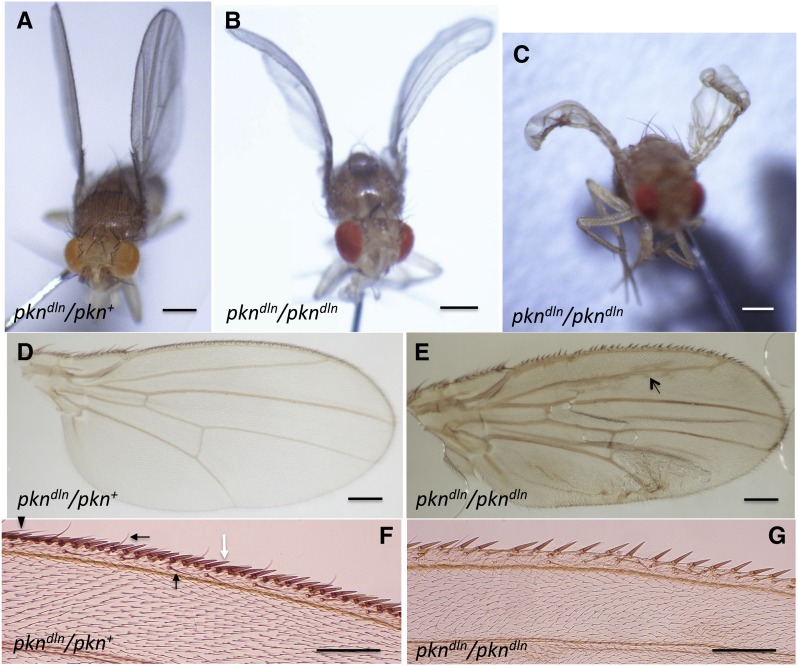
Characterization of the *delorean* wing mutation. (A) *w^1118^*; *pkn^dln^/pkn^+^* heterozygotes have normal wings. Scale bar = 0.35 mm. (B, C) *w^1118^*; *pkn^dln^/ pkn^dln^* homozygotes have wings that curve ventrally. The extent of curvature is variable. Bars = 0.35 mm. (D) Dissected wing of *w^1118^*; *pkn^dln^/ pkn^+^* heterozygote. Bar = 0.2 mm. (E) Dissected wing of *w^1118^*; *pkn^dln^/ pkn^dln^* homozygote shows creases from being mounted on a flat glass slide, longitudinal vein defects (arrow), and anterior margin defects. Bar = 0.2 mm. (F) Anterior wing margin of a *w^1118^*; *pkn^dln^/pkn^+^* heterozygote has regularly spaced stout bristles (white arrow), slender bristles (black arrowhead), and recurved bristles (black arrows). Bar = 0.1 mm. (G) The anterior wing margin of a *yw^1118^*; *pkn^dln^/ pkn^dln^* homozygote lacks many stout and recurved bristles and instead has gaps of bare marginal tissue. Bar = 0.1 mm.

In a screen of a second chromosome *P*-element insertion collection (Torok *et al.* 1993), we identified one line, *y w*; *P[lacW]k06808/CyO*, with a mutant phenotype that we named *delorean*. Flies homozygous for the *P[lacW]*k06808 insertion are unable to fly and their wings are held up from the thorax and curve ventrally (Figure 1, B and C), similar to the open doors of a Delorean automobile. The extent of curvature is variable, from a slight smooth curve to a severe folded-over phenotype. The anterior wing margin is also defective: there are gaps in marginal tissue, excessive spacing between stout mechanosensory bristles (Figure 1, E and G), and fewer sensillae and bristles relative to heterozygous siblings (Table 1). Occasionally, two or three stout bristles share a common socket, and sometimes a stout bristle appears in the margin tissue without a socket (Supporting Information, Figure S1A, Figure S1B, and Figure S1D). A total of 95% of homozygous *delorean* flies analyzed (n = 67) had one or more double-stout bristle clusters, and 15% had triple-stout bristle clusters. We also noted defects in wing veins in homozygous *delorean* flies such as ectopic vein material surrounding the longitudinal vein L2 (Figure 1E) and/or extending from the posterior crossvein (Figure S1C) (females 69.0%, SD 11.8%, n = 163 and males 56.9%, SD 18.7%, n = 116).

**Table 1:** Quantitative analysis of wing sensory structures

	Genotype				
	*w^1118^*	$\frac{pkn^{\mathrm{dln}}}{+}$	$\frac{pkn^{\mathrm{dln}}}{pkn^{\mathrm{dln}}}$	$\frac{pkn^{\mathrm{dln}};}{pkn^{\mathrm{dln}}}\frac{Dp\left(2;3\right)eve}{+}$	$\frac{pkn^{\mathrm{dln}}}{Df\left(2R\right)w45-30n}$
Twin sensillae	1.86 ± 0.38 (7)	2.0 ± 0 (8)	1.07 ± 0.7 (15)	2.0 ± 0 (7)	2.0 ± 0 (8)
	2.0 ± 0 (7)	2.0 ± 0 (8)	1.41 ± 0.61 (32)	1.83 ± 0.41 (6)	2.0 ± 0 (13)
Stout	77.6 ± 3.02 (8)	82.1 ± 2.17 (8)	48 ± 13.9 (15)	85.9 ± 1.64 (8)	82.0 ± 3.16 (10)
	73.8 ± 2.92 (8)	76.2 ± 3.22 (10)	53 ± 12.1 (32)	73.5 ± 6.16 (6)	74.3 ± 2.59 (13)
Ventral recurved	17.9 ± 0.9 (7)	18.0 ± 1.31 (8)	6.57 ± 2.9 (12)	17.3 ± 2.34 (6)	17.1 ± 1.45 (10)
	17.6 ± 1.99 (7)	18.0 ± 1.22 (9)	5.69 ± 2.35 (29)	15.5 ± 2.38 (4)	14.2 ± 2.33 (9)

Anesthetized adult flies were sorted by sex, wings were dissected and mounted flat, and twin campaniform sensillae, stout mechanosensory bristles, and ventral recurved chemosensory bristles were counted. Average number is given along with SD and number of wings scored (sample size). Because female wings are larger and have more bristles than males, we scored sexes separately. Top row for each trait gives values for females; bottom row for each trait gives values for males. Although it is not listed, all genotypes are in a *w^1118^* background. Wild-type wings have normally two twin campaniform sensillae (TCS) at the proximal end of longitudinal vein L1. Wings of flies homozygous for the *pkn^dln^* insertion have two, one, or zero TCS (female average ~57% of wild-type, *P* < 0.05; male average ~70% of wild-type, *P* < 0.05), ~66% of the wild-type number of stout bristles (*P* < 0.05), and ~33% of the wild-type number of ventral recurved bristles (*P* < 0.05). Flies homozygous for the *pkn^dln^* insertion that also carry a duplication of *pkn* show wild-type values of sensillae and bristles indicating a rescue of the *delorean* mutation. Flies hemizygous for the *pkn^dln^* insertion have wild-type values indicating the *pkn^dln^* allele is haplosufficient. *pkn*, *protein kinase N*.

A quantitative analysis focusing on specific anterior wing margin structures (Figure S1E) of the *delorean* wing phenotype was conducted (Table 1). Three of the traits analyzed showed significant differences between *delorean* homozygotes and either their heterozygous siblings or wild-type flies (*w^1118^*), regardless of sex. Reduced numbers of twin campaniform sensillae, stout bristles, and ventral recurved bristles were observed in *delorean* homozygotes (Table 1). We did not detect significant differences in the average number of slender bristles, dorsal recurved bristles, sensillae on vein L3 nor the anterior crossvein. Therefore, some sensory structures are affected by the *delorean* mutation whereas others are not. We consider the *delorean* wing phenotype to be a composite of a held-up position, ventral curvature, reduced sensory structures, and venation defects, suggesting that the affected gene plays diverse roles in wing development.

### The *delorean* phenotype results from a mutation in the *pkn* gene

**Figure 2:**
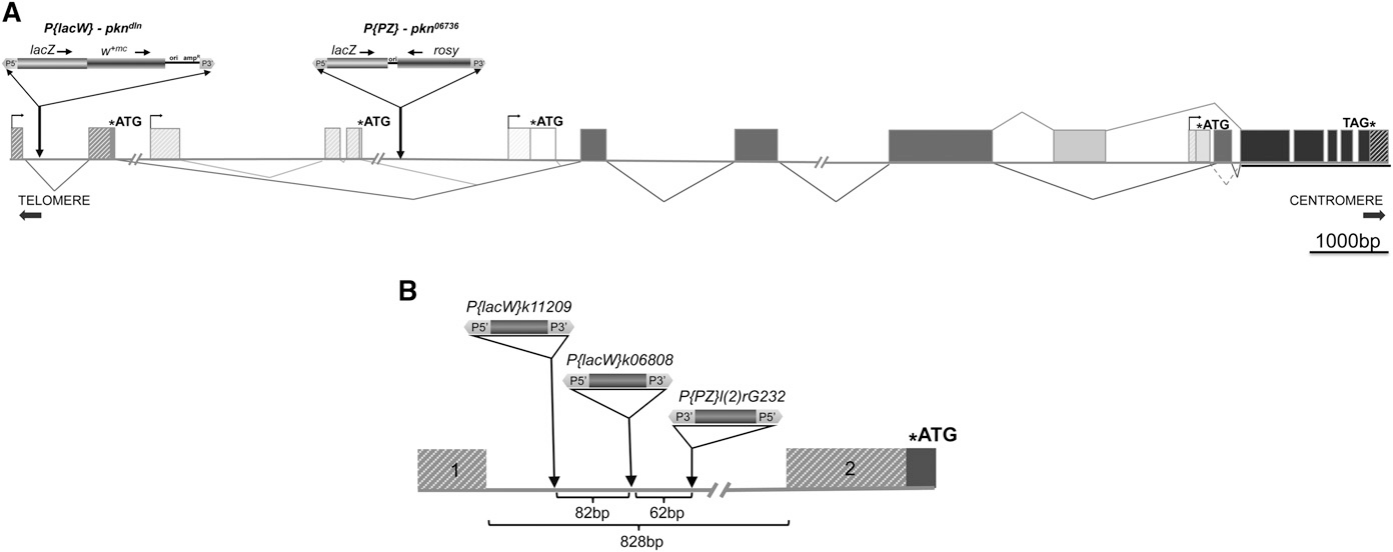
Molecular map of the *pkn* gene of *Drosophila melanogaster* and relative insertion positions of *pkn* alleles examined. (A) A molecular map of the *pkn* gene was generated using annotated data from Flybase (http://flybase.org/reports/FBgn0020621.html). We used all available annotated transcripts to compile the intron-exon structure of the *pkn* gene. We used the evidence ranking of all potential transcripts to estimate the most likely molecular structure of *pkn* with more strongly supported exons depicted with darker shading. There are four potential transcription start sites each with a unique translation start. Predicted exon splicing patterns for these transcripts are shown below the molecular structure and potential alternative splicing is shown above. All products of *pkn* transcription would be predicted to have exons that code for the kinase domain (darkest shading). The *pkn* gene encompasses approximately 22 kb and is oriented such that transcription is toward the centromere (minus orientation). In order to adjust the gene structure to a reasonable length, some introns are not shown in their entirety (intron gaps are noted with // marks). The position (in the fifth intron), of the previously characterized *P*-element insertion allele *pkn^06736^* described as an amorphic allele (Lu and Settleman 1999), is shown relative to the insertion of the *P[lacW]* element in intron 1 that causes the *delorean* mutation. (B) Enlargement of the region of intron 1 that is impacted by the *P*-element insertion alleles characterized in this study. All of these insertions are within a ~140-bp region of intron 1. The *pkn^k11209^* allele is caused by a *P[lacW]* insertion at genomic position 5172332 (8bp duplicated target ATCTGAGC); the *pkn^dln^* allele is caused by a *P[lacW]* insertion at genomic position 5172249 (8-bp duplicated target GTTTAACC); the *pkn^rG232^* allele is caused by an *P[PZ]* insertion at genomic position 5172186 (8-bp duplicated target GGCCGTGC).

To determine the cause of this interesting phenotype, we defined the insertion site of the *P[lacW]* transposon. Polytene chromosomes from the original *y w*; *P[lacW]k06808/CyO* stock were analyzed using *in situ* hybridization with a DNA probe containing sequence from the *mini-white* component of the *P[lacW]* transposon. The only hybridization signal found outside of the *white* gene on the *X* chromosome was at region 45C on chromosome 2 (data not shown). This indicates that the *P[lacW]* transposon is present only once in the *y w*; *P[lacW]k06808/CyO* genome. We next characterized the insertion site using gDNA from homozygous *y w*; *P[lacW]k06808* individuals. A total of 7 kb of gDNA flanking both sides of the *P[lacW]* insertion site was cloned. We sequenced three distinct clones and obtained 2.7 kb of genomic sequence flanking the *P[lacW]* insertion site. A BLAST search of the *Drosophila* genome identified this region as the 5′ end of the *pkn* gene, which maps to 45C. Sequence analysis revealed that the *P[lacW]* transposon is inserted in the first intron of *pkn* (at nucleotide position 5172249 of the *Drosophila* genomic sequence; http://www.flybase.org) and its orientation is identical to the transcribed strand (Figure 2A). Thus, *P[lacW]k06808* is an allele of the *pkn* gene, and we designate this allele as *pkn^dln^*.

To verify that this insertion causes the *delorean* phenotype, we outcrossed the original *y w*; *P[lacW]k06808/CyO* stock to *w^1118^*; *iso-2* for more than 40 generations and have maintained the *pkn^dln^* allele as an unbalanced stock by following the *mini-white* marker of the *P[lacW] transposon*. Flies from these stocks that are made homozygous for *pkn^dln^* allele display the complete *delorean* phenotype described previously. These findings indicate that the *delorean* phenotype is associated with the presence of the *P[lacW]* element in the *pkn* gene on the second chromosome and is not the result of other mutations in the genetic background of the original *P*-element insertion line from the Kiss collection. We also recovered 36 lines that had lost the *mini-white^+^* eye color marker associated with the *P[lacW]* transposon of *pkn^dln^* after a standard transposase-mediated mobilization procedure (see the *Materials and Methods*). When these lines were examined, most (34) exhibited a wild-type phenotype when heterozygous with either the *pkn^dln^* allele or a deficiency of the *pkn* gene region (data not shown), suggesting that there had been a precise excision of the *P[lacW]* transposon. This further confirms that the *delorean* phenotype is caused by the *P[lacW]* transposon insertion in the *pkn* gene and that loss of this insertion returns the phenotype to wild type. To see whether a wild-type allele of *pkn* and the surrounding region can rescue this phenotype, we used a duplication of cytological region 44B−46D on chromosome 3 (derived as a separable component of *Tp(2;3)eve^1.18^*; http://www.flybase.org). *pkn^dln^/pkn^dln^*; *Dp(2;3)eve^1.18^*/+ wings do not exhibit ventral curvature and flies no longer have anterior wing margin bristle defects or ectopic veins associated with the *delorean* phenotype (Figure S1F, Table 1), indicating that a single dose of region 45C suppressed these aspects of the *delorean* phenotype. Taken together, these results demonstrate that the *delorean* phenotype is due to the transposon insertion in *pkn*.

### The *delorean* phenotype is not a simple recessive, loss-of-function mutation

The previously characterized, loss-of-function *pkn^06736^* allele has a demonstrated multiphasic lethal phenotype (Lu and Settleman 1999), and we wanted to examine whether *pkn^dln^* also affected viability. The *delorean* mutation does not cause a reduction in viability when *pkn^dln^/pkn^dln^* offspring are compared with *pkn^dln^/pkn^+^* siblings from a cross of *pkn^dln^/pkn^+^* mothers to *pkn^dln^/pkn^dln^* fathers. We saw no significant difference from the expected equal proportions (47.3 ± 2.02% *pkn^dln^/pkn^dln^* offspring compared with 52.7 ± 2.02% *pkn^dln^/pkn^+^* offspring, *P >* 0.05). Thus, the *pkn^dln^* allele is adult viable, and its effect on the expression of the *pkn* gene must be fundamentally different than that of the *pkn^06736^* allele.

In our analysis of the composite *delorean* wing phenotype, we noted that wing defects are only seen in homozygous *pkn^dln^* flies. Heterozygous *pkn^dln^*/*pkn^+^* flies have wild-type wings (Figure 1, A, D, and F and Table 1). We wanted to further characterize the nature of the *delorean* mutation and examined the phenotype of the *pkn^dln^* allele when hemizygous with deficiencies that lack region 45C, as well as in combination with other alleles of *pkn*. Deficiency *Df(2R)w45-30n* lacks regions 45A-45E, thereby removing the *pkn* gene and fails to complement loss-of-function *pkn* alleles such as *pkn*^*06736*^, *pkn^2^*, and *pkn^3^*. Strikingly, the wing shape, anterior wing margin structures, and venation patterns of *pkn^dln^*/*Df(2R)w45-30n* flies were wild type (Figure S1G and Table 1), suggesting that the *pkn^dln^* allele is not a simple, recessive loss-of-function mutation. This was seen for all deficiencies of 45C examined [*Df(2R)Np3*, *Df(2R)Np5*, and *Df(2R)wun^GL^*; data not shown]. Furthermore, *pkn^dln^* complements the *pkn* alleles *pkn^06736^* and *pkn^2^* (Table 2 and data not shown). These data suggest that the *delorean* phenotype is recessive, as it takes two copies of the *pkn^dln^* allele to give the full *delorean* wing phenotype. In addition, we did see occasional *pkn^dln^*/*Df(2R)w45-30n* individuals that carry their wings held up from the thorax similar to *pkn^dln^* homozygotes (31.5% for males; 19.0% for females) consistent with the idea of a dose-sensitive, gain-of-function.

**Table 2:** Quantitative analysis of wing sensory structures in various genetic combinations of *pkn* alleles

	Genotype					
	$\frac{pkn^{\mathrm{dln}}}{pkn^{+}}$	$\frac{pkn^{\mathrm{dln}}}{pkn^{\mathrm{dln}}}$	$\frac{pkn^{k11209}}{pkn^{k11209}}$	$\frac{pkn^{rG232}}{pkn^{rG232}}$	$\frac{pkn^{\mathrm{dln}}}{pkn^{k11209}}$	$\frac{pkn^{\mathrm{dln}}}{pkn^{rG232}}$
Twin sensillae	2.0 ± 0 (8)	1.07 ± 0.7 (15)	1.36 ± 0.5 (11)	2.0 ± 0 (5)	0.45 ± 0.69 (11)	2.0 ± 0 (5)
	2.0 ± 0 (8)	1.41 ± 0.61 (32)	1.7 ± 0.48 (10)	1.86 ± 0.38 (7)	0.83 ± 0.41 (6)	1.17 ± 0.98 (6)
Stout	82.1 ± 2.17 (8)	48 ± 13.9 (15)	64.27 ± 6.32 (11)	55.9 ± 23.9 (7)	46.7 ± 5.8 (11)	70.8 ± 5.07 (5)
	76.2 ± 3.22 (10)	53 ± 12.1 (32)	63.2 ± 6.18 (9)	62.9 ± 12.8 (10)	49.8 ± 4.21 (5)	60.7 ± 11.3 (7)
Ventral recurved	18.0 ± 1.31 (8)	6.57 ± 2.9 (12)	8.58 ± 3.34 (12)	10.7 ± 2.8 (6)	nd*^a^*	12 ± 1.73 (3)
	18.0 ± 1.22 (9)	5.69 ± 2.35 (29)	11.5 ± 3.42 (8)	11.7 ± 2.24 (9)	nd*^a^*	11 ± 3.16 (7)

	Genotype				
	$\frac{pkn^{\mathrm{dln}}}{pkn^{06736}}$	$\frac{pkn^{k11209}}{pkn^{06736}}$	$\frac{pkn^{\mathrm{dln}}}{pkn^{\mathrm{dln}\Delta 5}}$	$\frac{pkn^{\mathrm{dln}\Delta 5}}{pkn^{\mathrm{dln}\Delta 5}}$	$\frac{pkn^{\mathrm{dln}\Delta 5}}{Df\left(2R\right)w45-30n}$
Twin sensillae	1.89 ± 0.33 (9)	2 ± 0 (10)	0.86 ± 0.38 (7)	1.5 ± 0.71 (2)	2.0 ± 0 (7)
	1.89 ± 0.33 (9)	1.6 ± 0.5 (7)	1.29 ± 0.49 (7)	1.67 ± 0 0.58 (3)	1.8 ± 0.45 (5)
Stout	81.2 ± 3.36 (10)	76.6 ± 5.48 (10)	59.2 ± 5.07 (9)	75.8 ± 17.2 (4)	85.5 ± 2.35 (6)
	77.6 ± 6.38 (10)	68.75 ± 5.85 (8)	60.3 ± 3.44 (6)	82.5 ± 1.29 (4)	78.0 ± 1.58 (5)
Ventral recurved	20 ± 1.83 (10)	20.8 ± 1.47 (10)	7.67 ± 2.83 (9)	14.5 ± 3.42 (4)	19.1 ± 0.9 (7)
	17.5 ± 0.84 (6)	17.6 ± 1.6 (8)	11.8 ± 3.06 (6)	19.5 ± 1.73 (4)	13.5 ± 4.95 (2)

Format is the same as in Table 1. Average number is given along with SD and number of wings scored (sample size). Top row gives values for females; bottom row gives values for males. All genotypes are in a *w^1118^* background except for *pkn^rG232^* homozygotes, which are in a *ry*^*506*^ background. The *P*-element insertion alleles, *pkn^dln^*, *pkn^k11209^*, and *pkn^rG232^*, are located in the same intron of the *pkn* gene (see Figure 2). Homozygotes of the three insertions show the *delorean* phenotype although to a variable extent. *pkn*, *protein kinase N*.

a nd = not determined; could not be scored unequivocally because wings were too distorted.

To further elucidate the nature of the *pkn^dln^* allele we decided to look at the phenotypic consequence of reducing *pkn* expression in wing tissue. We used the GAL4/UAS ectopic expression system to drive tissue-specific production of a *pkn* double-stranded RNA that would specifically degrade *pkn* messenger RNA by RNA interference. Several different wing-specific GAL4 drivers were examined (Table S1), each in combination with the same UAS-*pkn-RNAi* transgene on the third chromosome. This UAS-*pkn-RNAi* construct causes lethality when a GAL4 driver that is ubiquitously expressed due to the presence of either the α *tubulin* or *actin5C* promoter is used (data not shown). This is expected, given the multiphasic lethality associated with the null allele, *pkn^06736^* (Lu and Settleman 1999), and indicates that loss of *pkn* RNA can be achieved using this system. The reduction of *pkn* using wing-specific GAL4 drivers does not generate a mutant phenotype (Figure S2). All wings examined exhibit wild-type anterior wing margin bristle numbers consistent with values reported for Canton-S (Table S2; Hartenstein and Posakony 1989). For this reason, we are able to rule out the possibility that the *delorean* phenotype is due to a loss of function mutation in the *pkn* gene.

### The *delorean* phenotype is shared by other transposon insertions in the first intron of *pkn*

There are several transposon insertions in region 45C (Figure 2B) for which stocks are available. Two of these transposon insertions, *P[lacW]l(2)k11209* and *P[PZ]l(2)rG232*, are in close proximity to *P[lacW]k06808* within a ~140-bp region of the first intron of the *pkn* gene. We designate these alleles as *pkn^k11209^* and *pkn^rG232^*, respectively (*l(2)k11209* and *l(2)rG232* were mapped to 45C in Spradling *et al.* 1999; we mapped *l(2)rG232* to genomic position 5172186). We note that the insertions of *P[lacW]l(2)k11209* in *pkn^k11209^* and *P[lacW]k06808* in *pkn^dln^* are in the same orientation such that the 5′ terminus and 3′ terminus of the *P[lacW]* element have the same relative orientation as transcription from the *pkn* gene (Figure 2A). The *P*-element of *P[PZ]l(2)rG232* is in the opposite orientation. Individuals homozygous for the *pkn^k11209^* and *pkn^rG232^* alleles exhibit a similar *delorean* wing phenotype, and all are able to complement null alleles of the *pkn* gene (Table 2). Both *pkn^k11209^* and *pkn^rG232^* fail to complement *pkn^dln^* as heterozygous individuals have a *delorean* wing phenotype similar to that of *pkn^dln^* homozygotes (Table 2). We conclude that these *P*-element insertion alleles exhibit a *delorean* phenotype because of a similar disruption of *pkn* gene expression. In addition, the close proximity of the insertion sites indicates that the relative location of the insertions within the first intron is a more important determinant of the mutant phenotype than the type and orientation of the transposon.

### A deletion derivative of the *P[lacW]k06808* insertion exhibits a less-severe *delorean* phenotype

Most of the transposase-induced excisions of the *P[lacW]* element of *pkn^dln^* resulted in reversion to wild type due to complete loss of the *P*-element. However one excision event was imprecise and generated a line (designated *pkn^dlnΔ5^*) that had lost the *mini-white^+^* eye color marker associated with *P[lacW]*. This *pkn^dlnΔ5^* derivative was found to have a milder *delorean* phenotype when heterozygous with *pkn^dln^* (Table 2). gDNA was extracted from individuals homozygous for *pkn^dlnΔ5^* and the region surrounding the *P[lacW]* insertion site was amplified by PCR. DNA sequencing of the PCR product revealed that the *pkn* gDNA and most of the *P[lacW]* element was intact, but a 1874-bp deletion had removed part of the *mini-white^+^* cassette of *P[lacW]*, hence its white-eyed phenotype.

**Figure 3:**
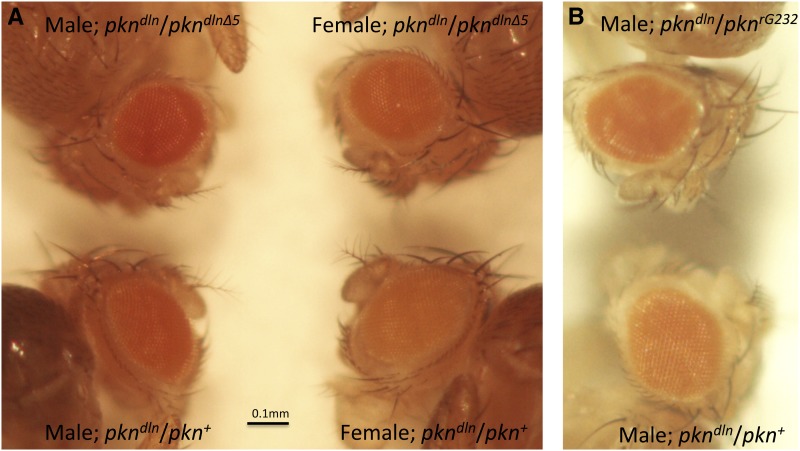
Expression of the *mini-white^+^* reporter of *pkn^dln^* is influenced by the *pkn* insertion allele in *trans*. (A) Eye phenotypes of 7- to 10-day-old flies are shown. Males are shown on the left, females are shown on the right. Flies on top are genotype *pkn^dln^*/*pkn^dlnΔ5^* whereas flies on the bottom are *pkn^dln^*/*pkn^+^*. Despite the sex-specific differences in expression of the *mini-white^+^* reporter, it can be seen that even though all flies contain only one dose of the *mini-white^+^* reporter, flies with the genotype *pkn^dln^*/*pkn^dlnΔ5^* have increased pigmentation. (B) This same increase in pigmentation can be seen even when the *pkn* insertion allele in *trans* lacks sequence in common with the *mini-white^+^* reporter of *pkn^dln^*. Five-day-old males with the genotype *pkn^dln^*/*pkn^rG232^* (top) or genotype *pkn^dln^*/*pkn^+^* (bottom) are shown.

We found that homozygous *pkn^dlnΔ5^* individuals have a less severe phenotype than *pkn^dln^* homozygotes (*P* < 0.05, Table 2). However the *pkn^dlnΔ5^* allele behaves similarly in its interaction with either the loss-of-function allele *pkn^06736^* or with *Df(2R)w45-30n* in heterozygous flies, resulting in a wild-type wing phenotype (Table 2). This deletion derivative of the *P[lacW]k06808* insertion also behaves as a recessive mutation, with the only difference between *pkn^dlnΔ5^* and the original *pkn^dln^* allele being a reduction in the severity of the wing phenotype (Table 2). Of particular note with respect to the *pkn^dlnΔ5^* allele is its ability to increase the expression of the intact *mini-white^+^* marker of *P[lacW]* present in *pkn^dln^* when flies that are heterozygous for these alleles are examined (*pkn^dlnΔ5^*/*pkn^dln^*, Figure 3A). This “*trans*-allelic” interaction does not require the presence of extensive homology between paired transposons or a precisely shared insertion site. This conclusion is based on the elevated expression of the *mini-white^+^* marker of *pkn^dln^* when heterozygous with *pkn^rG232^* (Figure 3B), an allele caused by the insertion of the *P[PZ]* transposon that carries the *rosy* gene as a marker and contains only the *P*-element ends and the *lacZ* gene in common with *P[lacW]*.

### Molecular analysis of expression from the *delorean* allele

**Figure 4:**
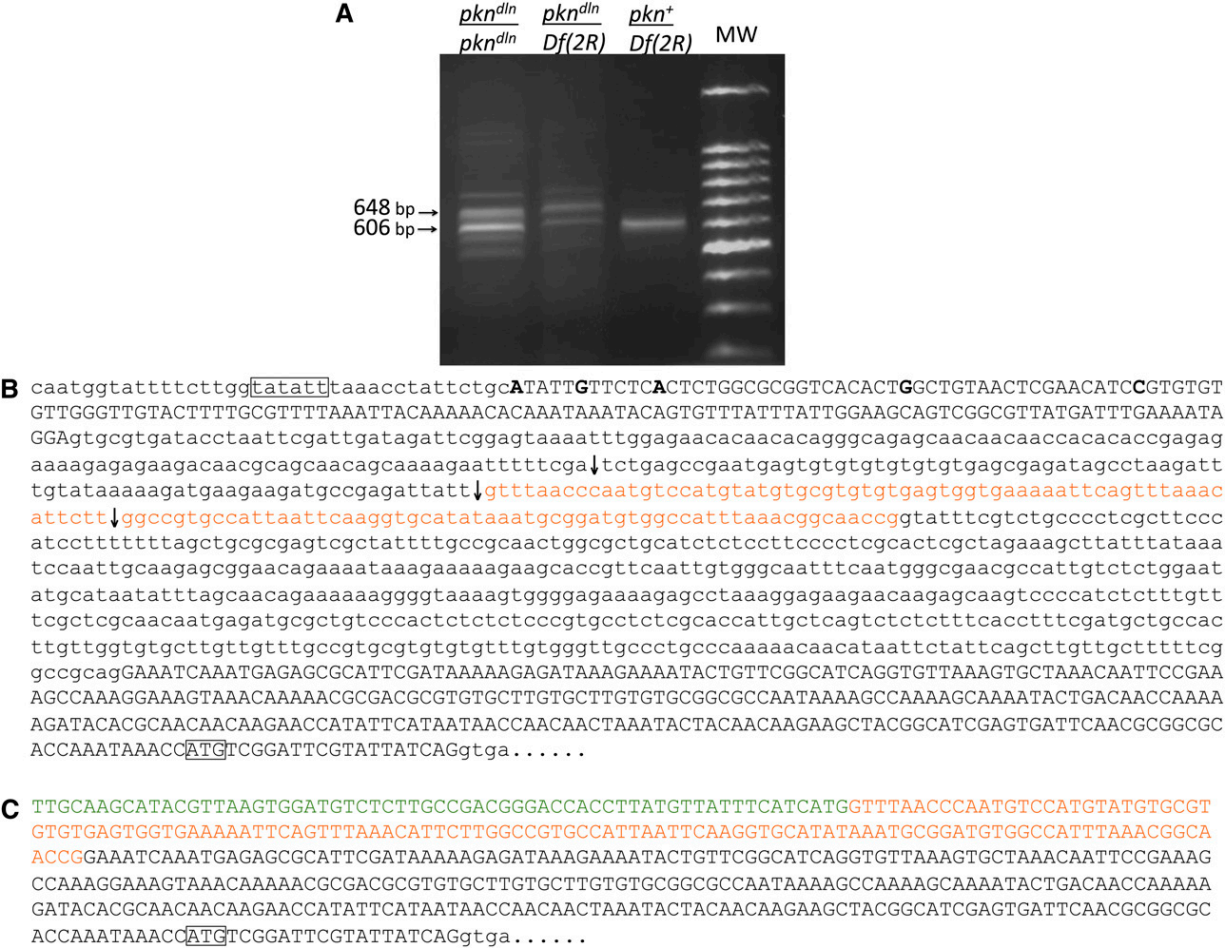
The *pkn^dln^* allele generates a novel transcript. (A) Rapid amplification of 5′ cDNA ends (5′ RACE) products derived from pupal RNA. Left to right: *pkn^dln^*/*pkn^dln^* homozygous products; *pkn^dln^*/*Df(2R)w45-30n* hemizygous products; *pkn^+^*/*Df(2R)* hemizygous product; 100-bp molecular weight standard (Promega). (B) Sequence of the 5′ end of the *pkn* transcriptional unit. Only one strand of DNA is shown. Exonic bases are uppercase; nonexonic bases are lowercase. The putative TATA box is boxed as is the predicted translational start site. The 5′-most nucleotide of various known *pkn* cDNAs are bolded. The positions of the three intron 1 *P-element* insertions described in this article are indicated with arrows (top to bottom: *pkn^k11209^*, *pkn^dln^*, *pkn^rG232^*). Intronic bases that are included in the *pkn^dln^*-specific transcript are orange. (C) Sequence of the 5′ end of the *pkn^dln^*-specific transcript. Exonic bases are uppercase; intronic bases are lowercase. The portion derived from the 3′ end of the *P[lacW]* transposon is highlighted in green. Bases that are excised from *pkn*^+^ transcripts but are included in the *pkn^dln^* transcript (corresponding to the orange sequence shown in part 4B) are highlighted in orange. The predicted translational start site is boxed.

To better understand the molecular basis of the *delorean* phenotype, we wanted to determine the nature of transcription from the *pkn^dln^* allele. An examination of the modENCODE Temporal Expression Data (flybase; http://flybase.org/reports/FBgn0020621.html) informed our choice of the white pupae stage as the source of RNA to be analyzed. We reasoned that this stage of development had moderate levels of expression and would also be undergoing significant development of the wing discs. 5′ RACE on total RNA isolated from 1-d-old pupae resulted in two products in *pkn^dln^* homozygotes and *pkn^dln^*/*Df(2R)w45-30n* hemizygotes (Figure 4A). The smaller of the two products was found to comigrate with the wild-type product. Sequencing of the RACE products revealed that this smaller *pkn^dln^* product is equivalent to the wild-type transcript (Figure 4B), demonstrating that the intron containing the *P*-element insertion can be recognized and excised in both *pkn^dln^* homozygotes and *pkn^dln^*/*Df(2R)w45-30n* hemizygotes. Sequencing of the larger of the two *pkn^dln^*-derived RACE products indicates that transcription from the *pkn^dln^* allele initiates within the 3′ end of the *P*-element (*P3*′; Figure 4C). The 5′ end of the *delorean*-specific transcript begins with 63 nucleotides derived from the *P3′* end of *P[lacW]*, followed by 122 nucleotides of the first intron immediately downstream of the insertion site that are then spliced to the 5′ end of exon 2. Thus, the *pkn* transcripts in *pkn^dln^* flies represent a mixture of wild-type and aberrant messenger RNAs. The protein made using the *delorean*-specific transcript is predicted to be identical to that made by the wild-type transcripts. These results confirm our genetic analyses characterizing the *delorean* phenotype as a gain-of-function mutation.

### The *delorean* phenotype can be enhanced by loss of JNK pathway components

**Figure 5:**
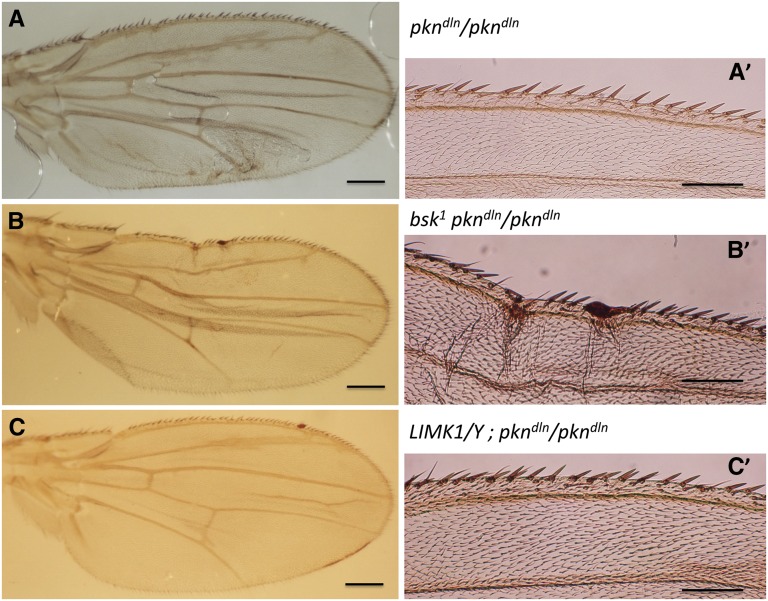
The *delorean* mutation interacts with *basket* but not with *LIMK1*. (A, A′) Dissected wing of *pkn^dln^/ pkn^dln^* shows the *delorean* phenotype. (B, B′) Dissected wing of *bsk^1^ pkn^dln^/ pkn^dln^* shows a more severe *delorean* phenotype. (C, C′) Dissected wing of *LIMK1^EY08757^/LIMK1^+^*; *pkn^dln^ / pkn^dln^* shows the *delorean* phenotype. See Table 3 for quantitative analysis. Left panels (A, B, C) are whole wings, scale bar = 0.2 mm. Right panels (A′, B′, C′) are close-ups of anterior wing margins, bar = 0.1 mm.

To better understand the cell biological basis of the *delorean* phenotype, we examined its genetic interaction with mutations that have been previously identified to interact with the *pkn* loss-of-function allele *pkn^06736^* during embryonic development. We looked specifically at the *Drosophila* homolog of mammalian Jun-N-terminal kinase (D-JNK), encoded by *bsk*. The loss-of-function *bsk^1^* allele increased the frequency of embryos with dorsal closure defects derived from *pkn^06736^* germline clones (Lu and Settleman 1999). We found that the *delorean* wing phenotype is enhanced upon reduction of D-JNK when we observed the anterior wing margin of *delorean* homozygotes that carry one copy of the *bsk^1^* allele (genotype: *bsk^1^ pkn^dln^* / *pkn^dln^*; Figure 5). Compared with *pkn^dln^*/*pkn^dln^* homozygotes alone, the average numbers of twin sensillae (0.43 *vs.* 1.07), stout bristles (42.5 *vs.* 48), and ventral recurved bristles (5.86 *vs.* 6.57) in females are reduced in *bsk^1^ pkn^dln^* / *pkn^dln^* flies (see Table 3). This suggests that the *delorean* mutation causes a disruption in some aspect of wing morphogenesis that may be related to a function also mediated by the JNK pathway.

**Table 3:** Quantitative analysis of wing sensory structures of *pkn^dln^* homozygotes in combination with *basket* or *LIMK1* indicate that *bsk* can enhance the severity of the *delorean* mutation

	Genotype		
	$\frac{pkn^{\mathrm{dln}}}{pkn^{\mathrm{dln}}}$	$\frac{bsk^{1}pkn^{\mathrm{dln}}}{pkn^{\mathrm{dln}}}$	$\frac{LIMK1^{EY08757}}{LIMK1^{+}\;or\;Y};\frac{pkn^{\mathrm{dln}}}{pkn^{\mathrm{dln}}}$
Twin sensillae	1.07 ± 0.7 (15)	0.43 ± 0.51 (14)	1 ± 0 (6)
	1.41 ± 0.61 (32)	0.75 ± 0.75 (12)	1.33 ± 0.82 (6)
Stout	48 ± 13.9 (15)	42.5 ± 11.9 (20)	53.6 ± 8.92 (7)
	53 ± 12.1 (32)	42.4 ± 8.23 (13)	57.3 ± 3.67 (6)
Ventral recurved	6.57 ± 2.9 (12)	5.86 ± 1.95 (7)	4.25 ± 2.22 (4)
	5.69 ± 2.35 (29)	4.18 ± 1.94 (11)	7.17 ± 1.17 (6)

Format is same as in Table 1 and Table 2. Flies that are heterozygous for a loss-of-function allele of *basket* (*bsk*^*1*^) in a *pkn^dln^* homozygous background have on average significantly fewer twin campaniform sensillae than flies that are homozygous for the *pkn^dln^* insertion alone and flies heterozygous for a loss of function allele of *LIMK1* in a *pkn^dln^* homozygous background (*P* < 0.05). The same significant reduction is found when comparing average number of stout mechanosensory bristles in male flies but not in females. Average number of ventral recurved bristles was significantly different between *bsk*^*1*^ *pkn^dln^ / pkn^dln^* and LIMK1/+; *pkn^dln^* / *pkn^dln^* flies (*P* < 0.05) but not between *bsk*^*1*^ *pkn^dln^ / pkn^dln^* and *pkn^dln^* / *pkn^dln^* flies. Data were analyzed by Kruskal-Wallis tests followed by Tukey’s HSD.

**Figure 6:**
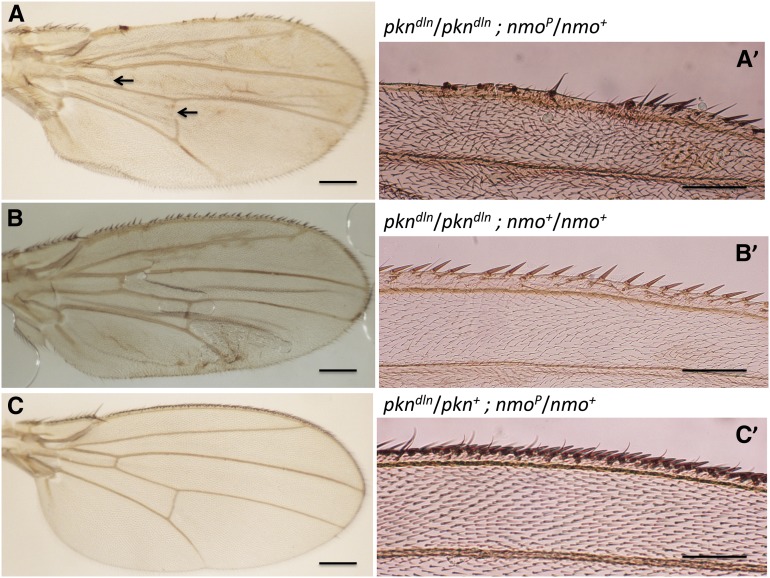
The *delorean* mutation interacts with *nemo*. (A, A′) Dissected wing of *pkn^dln^/ pkn^dln^* ; *nmo^P^*/+ shows a more extreme *delorean* phenotype than wings of *pkn^dln^* siblings without *nmo^P^*. Gaps in crossveins are indicated by arrows. (B, B′) Dissected wing of *pkn^dln^/ pkn^dln^* shows the *delorean* phenotype. (C, C′) Dissected wing of *pkn^dln^/pkn^+^* ; *nmo^P^/nmo^+^* shows a wild-type phenotype. Left panels (A, B, C) are whole wings, scale bar = 0.2 mm, and right panels (A′, B′, C′) are close-ups of anterior wing margins, bar = 0.1 mm.

The parallel nature of the interaction between a component of the JNK signaling pathway and the *pkn^dln^* and *pkn^06736^* alleles as observed in two different morphogenetic processes prompted us to examine other participants in JNK signaling. We examined the *Drosophila Nemo-like kinase* (*nmo*) because of its potential role in the integration of signaling pathways involved in regulation of wing patterning (*wg* and *dpp* signaling) with JNK-mediated programmed cell death (Mirkovic *et al.* 2002). Defects associated with the anterior wing margin and wing crossveins are enhanced upon reduction of Nmo (Figure 6A, genotype: *pkn^dln^*/*pkn^dln^*; *nmo^P^*/*nmo^+^*). We found that 50% of flies with the genotype *pkn^dln^*/*pkn^dln^*; *nmo^P^*/*nmo^+^* exhibit complete or partial loss of anterior or posterior crossveins, or both. Loss of crossvein structure is not typically seen in *pkn^dln^*/*pkn^dln^* flies but is a reported consequence of expressing *nmo* in the wing using an epidermal Gal4 driver (Verheyen *et al.* 2001; Mirkovic *et al.* 2002). The extreme loss of anterior wing margin material in *pkn^dln^*/*pkn^dln^*; *nmo^P^*/*nmo^+^* flies made it difficult to complete our standard quantitative analysis without a bias in the samples analyzed. Nonetheless, it is clear that a reduction in the level of Nmo dramatically affects the *delorean* phenotype and our analysis of the wings from *pkn^dln^*/*pkn^dln^*; *nmo^P^*/*nmo^+^* individuals suggests that the balance between JNK signaling and signaling pathways involved in patterning of the wing is also compromised in *pkn^dln^* homozygotes.

We also wanted to explore interactions with Rho pathway components that have been found to be involved in wing morphogenesis. Expression of a kinase-deficient form of LIM-kinase 1 (LIMK1) results in wing and leg malformations that can be suppressed if the levels of Rho1 are reduced (Chen *et al.* 2004). This genetic interaction is not seen with PKN however as the loss-of-function allele *pkn^06736^* does not have the same ability to suppress the effects of the kinase-deficient LIMK1 (Chen *et al.* 2004). We examined whether *LIMK^EY08757^* affected the *delorean* phenotype using flies of the genotype *y^1^w^67c23^P{EPgy2}LIMK1^EY08757^*; *pkn^dln^ / pkn^dln^*. We did not find a significant difference in phenotype relative to *pkn^dln^/pkn^dln^* homozygotes (Figure 5 and Table 3). This informs us that PKN’s effector function is not likely to be related to that of LIMK1.

## Discussion

### Characterization of the *pkn^dln^* allele

We have characterized the *delorean* mutation that causes defects in wing morphology due to a *P[lacW]* insertion in the *protein kinase N (pkn)* gene of *Drosophila melanogaster*. An essential function of the *pkn* gene is not disrupted by the *P[lacW]* insertion because individuals homozygous for the *pkn^dln^* allele can be recovered in expected numbers. Although the wing phenotype of heterozygous *pkn^dln^/pkn^+^* individuals is wild type in all respects, the effect of the transposon insertion cannot be classified as a loss-of-function mutation. This is shown by the wild-type wing morphology of individuals that are hemizygous for the *pkn^dln^* allele (*pkn^dln^*/*Df(2R)w45-30n*) and because the reduction of *pkn* RNA in wing tissue using RNA interference has no phenotypic consequence. Moreover, it is clear that the *delorean* wing phenotype is only seen when both alleles present contain a transposon insertion (or a derivative of this transposon) within a 140-bp region of the first intron of the *pkn* gene. For these reasons, we consider the *delorean* phenotype to be caused by a recessive mutation.

A model of the molecular structure of the *pkn* gene based on annotated transcripts that have been identified to date indicates that there are a number of alternative transcription start sites as well as alternate exon usage (Figure 2A; http://www.flybase.org). Of the four predicted alternative transcription start sites, there are three that would generate a transcript capable of encoding the amino-terminal domain of PKN required for both Rho binding and the ability to rescue the loss-of-function *pkn^06736^* allele (Lu and Settleman 1999). The position of the *P[lacW]* insertion of *pkn^dln^* reflects the bias that *P*-elements have for insertion near promoters and 5′ regulatory regions (Berg and Spradling 1991) and would likely have an impact on one of the more well-supported transcripts (indicated by darker shading in Figure 2A) that uses the 5′ most transcription start site. Our molecular analysis of the *delorean* allele using 5′ RACE aimed to determine how a transposon insertion within the first intron of the *pkn* gene could generate a mutant phenotype. The presence of *P[lacW]* does not prevent the proper splicing of the first two *pkn* exons and indicates that the predicted, wild-type transcript can be generated using the 5′ most transcription start site. Our 5′ RACE analysis also identified a transcript specific to the *delorean* allele that is the result of initiation from within the 3′end of the *P[lacW]* transposon and includes intron sequence spliced to the second exon of the *pkn* gene. The predicted PKN protein produced from the *delorean* transcript is the same as that from the wild-type transcript. Given our determination that the composite *delorean* phenotype is not the result of a loss-of-function mutation, it is reasonable to assume that the identified *delorean*-specific transcript generated from the *pkn^dln^* allele is the cause of the mutant phenotype. Our finding that reducing the levels of *pkn* transcript using RNAi has no effect on wing morphology is in support of this conclusion. For these reasons it is likely that the *delorean* phenotype is due to altered regulation or ectopic expression of the *delorean*-specific transcript. A similar situation has been described previously (LaFave and Sekelsky 2011) whereby initiation of transcription was found to occur from within a *P*-element. The authors conclude that an outcome of such cryptic transcription would likely be altered expression of the gene under study.

Our genetic analysis indicates that the pairing context of the *pkn^dln^* allele is an important determinant of the *delorean* phenotype. This is demonstrated by our observations of the *pkn^dlnΔ5^* allele, an imprecise excision derivative of *pkn^dln^* that has a deletion within the *mini-white* gene marker of *P[lacW]* and is as a result white-eyed. Expression of the intact *mini*-*white* reporter in the *pkn^dln^* allele is elevated in *pkn^dln^*/*pkn^dlnΔ5^* heterozygotes as revealed by their eye phenotype, which is darker than *pkn^dln^*/*pkn^+^* individuals. Transcription from the *mini-white* gene is correlated with the degree of pigmentation (Silicheva *et al.* 2010). An increase in expression of the *mini-white* reporter may reflect the creation of a local chromosomal environment that is favorable for transcription in this heteroallelic state due to the presence of paired insertion-bearing alleles. The responsiveness of the *mini-white* gene to the chromatin environment is well known (Bier *et al.* 1989; Zhang and Odenwald 1995). It follows that the paired state of the *delorean* allele (or its derivative) as is found in *pkn^dln^/pkn^dln^*, represents an optimized conditions for expression of both the *mini*-*white* reporter and the *delorean*-specific *pkn* transcript that generates the mutant wing phenotype. We consider it reasonable that the *delorean* phenotype in its entirety results when maximum levels of the *delorean*-specific transcript are expressed and that this in turn is more likely to occur with the pairing of insertion-bearing alleles.

**Figure 7:**
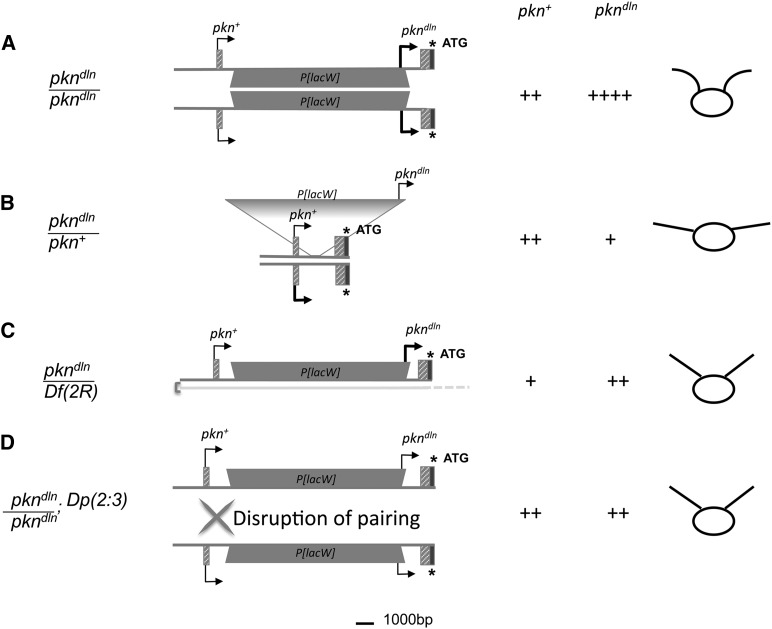
Pairing dependence of *pkn^dln^* expression as a working model to explain the *delorean* phenotype. The model depicts the 5′ end of the *pkn* gene showing only the affected region of the gene (first two exons and intron 1; refer to Figure 2). Thickness of arrows represents assumed relative levels of transcription. Transcription of the wild-type transcript (*pkn^+^*) begins at the first exon. Initiation of the *delorean*-specific transcript, referred to as *pkn^dln^*, from within the *P*-element generates a transcript that is either expressed ectopically or regulated differently than *pkn^+^*. Relative levels of *pkn^+^* and *pkn^dln^* transcripts are given with the “+” symbol. The schematic in the far right column depicts the position and shape of the wing for each genotype. (A) A reduction in the expression of wild-type *pkn* and an increase in the expression of *pkn^dln^*, occurs when the *pkn^dln^* allele is homozygous. The level of *pkn^dln^* expression is elevated as a result of pairing thereby causing the composite wing defects of the *delorean* phenotype. The level of wild-type transcript may be reduced due to the additional sequence in the form of the *P*-element in the first intron that must be removed by splicing. (B) Levels of the *delorean*-specific transcript are reduced in *pkn^dln^*/*pkn^+^* heterozygotes due to the absence of *P[lacW]* in *trans* to the *pkn^dln^* allele. The reduction in the level of *pkn^dln^* transcript is below a threshold needed to generate a phenotype. (C ,D) Our analysis of heteroallelic combinations of *pkn* alleles also lead us to the conclusion that a reduction in the level of *delorean*-specific transcript occurs in the absence of either any homology-based pairing in *trans* (C; *pkn^dln^*/*Df(2R)*) or when pairing is disrupted (D; *pkn^dln^*/*pkn^dln^*; *Dp(2:3)*). In the case of *pkn^dln^*/*Df(2R*) there would be a reduction in the level of both *pkn^dln^* and *pkn^+^* transcripts. Reduced levels of *pkn^dln^* transcript can suppress the *delorean* phenotype. In the case of *pkn^dln^*/*pkn^dln^*; *Dp(2:3)*, the transposed sequence present in the duplication (*Dp)* is able to disrupt pairing between the *pkn^dln^* alleles due to homology resulting in decreased *pkn^dln^* transcript levels. It is also likely that the level of wild-type *pkn* transcription is affected by the pairing state at the *pkn* gene. We consider it reasonable to infer that the relative levels of a *delorean*-specific transcript are involved in determining the extent of the *delorean* phenotype.

We have not used chromosomal rearrangements to specifically disrupt pairing and test for classic transvection effects in our analysis of the *delorean* phenotype. However, we do see suppression of the *delorean* phenotype in *pkn^dln^/pkn^dln^*; *Dp(2:3)eve^1.18^* individuals that are wild type in all respects except for the held up position of their wings (also seen in *pkn^dln^* homozygotes). The lack of rescue by an additional copy of the *pkn* gene present in *Dp(2:3)eve^1.18^* is not unexpected in the case of a mutant phenotype caused by misexpression of a *pkn* transcript, as we have argued to be the case for the *pkn^dln^* allele. In fact the observation that there is suppression at all argues the amount of misexpressed *pkn* has been reduced. The presence of an additional homologous pairing partner could influence the interaction between the *pkn^dln^* alleles. Somatic pairing of homologous chromosomal regions is robust in *Drosophila* (Apte and Meller 2012) and the local chromosomal interactions that result are not only common (Chen *et al.* 2002; Mellert and Truman 2012) but with profound influence on expression (Sass and Henikoff 1999; Wu and Morris 1999; Ou *et al.* 2009). We postulate that in an unpaired state the *pkn^dln^* allele in *pkn^dln^/pkn^dln^*; *Dp(2:3)eve^1.18^* individuals would still be able to express the *delorean*-specific transcript, albeit at lower levels due to asynapsis (Goldsborough and Kornberg 1996), and thereby with reduced phenotypic impact (*i.e.*, result in a held-up wing phenotype but a wild-type anterior wing margin, for example). The *pkn^dln^* allele would also be in an “unpaired” state when hemizygous with a deficiency of the *pkn* gene. We observed that wings of *pkn^dln^*/*Df(2R) w45-30n* hemizygotes are also on occasion held up vertically and a similar phenotype has been reported for *pkn^rG232^*/*Df(2R)wun^GL^* hemizygotes (Zhang *et al.* 1996). The absence of the *pkn* gene region in *trans* to the insertion-bearing *pkn^dln^* chromosome may favor a change in the topology of the region such that the transcription of a wild-type *pkn* product is favored over that of the *delorean*-specific transcript. Changes in topology have also been suggested to mediate the “enhancer bypass” mechanism underlying specific cases of transvection (Morris *et al.* 1998). The “held-up” wing phenotype seen in *pkn^dln^/pkn^dln^*; *Dp(2:3)eve^1.18^* and *pkn^dln^*/*Df(2R)* individuals would then reflect a sensitivity to low levels of misexpression from the *pkn^dln^* allele that persists in an unpaired state (see Model, Figure 7). Thus we consider the *delorean* wing phenotype to be due to a pairing-dependent, recessive mutation that behaves as a dose-sensitive, gain-of-function due to misexpression.

### Comparative analysis of the *delorean* phenotype

We have described the wing phenotype associated with the *pkn^dln^* allele as a composite of several morphological defects. Changes in the overall position and structure of the wing are seen in *delorean* homozygotes as a held up wing and a ventral curvature to the wing, respectively. The flightless, held up wing phenotype of *delorean* flies resembles that of known *Drosophila* mutants with defects in flight muscles (*vertical wings*, Deak 1977; *upheld*, Deak *et al.* 1982; *wings up A*—a mutation in the muscle protein troponin, Barbas *et al.* 1993). The curved wing phenotype of *pkn^dln^* homozygotes suggests a disruption of epithelial cell morphology or changes in cell growth that would alter the relationship between the dorsal and ventral surfaces of the adult wing. Also associated with the *delorean* phenotype is a reduction in sensory structures and venation defects. The loss of a subset of anterior wing margin bristles suggests a patterning defect whereby external sensory organ structures are transformed to internal structures. Errors in patterning could also explain the ectopic veins formed in *delorean* mutants as epithelial cells of the developing wing disc fail to make the appropriate choice between a vein or intervein fate. Such a varied response within a single tissue provides a unique opportunity to establish a mechanism by which misexpression of *pkn* mediates these apparently diverse effects and in turn determine the genetic interactions that are required for PKN involvement in wing morphogenesis.

Previous studies of the *pkn* gene in *Drosophila* have used a loss-of-function mutation that is the result of a *P[PZ]* transposon insertion in the fifth intron of the *pkn* gene generating the embryonic lethal, *pkn^06736^* allele (Lu and Settleman 1999; see Figure 2A). The embryonic lethality of *pkn^06736^* can be rescued with a *pkn* cDNA that is expressed using a heat shock promoter (Betson and Settleman 2007). Rescued individuals exhibit a mild adult wing phenotype that exhibits considerable variability that may reflect a functional redundancy in PKN function at later stages of development. The absence of a loss-of-function phenotype due to functional redundancy has been argued to be the case for at least 60% of genes in *Drosophila* (Miklos and Rubin 1996) and places constraints on the use of the *pkn^06736^* allele in an analysis of PKN function. For this reason, our analysis of the *delorean* wing phenotype was undertaken to better understand the molecular lesion associated with *pkn^dln^* as it represents a unique allele of *pkn* that is adult viable and not the result of a loss-of-function mutation. Furthermore, our characterization of the molecular structure of *pkn^dln^* indicates that this particular allele represents an opportunity to study the effects of misexpression of the *pkn* gene.

That the *delorean* phenotype represents a misexpression of PKN in affected tissues is supported by the observation of Betson and Settleman (2007) that overexpression of the kinase domain of PKN specifically in the wing margin generates a wing phenotype that is analogous to the anterior wing margin defects of *delorean* flies. Loss of sensory bristles along the anterior wing margin as well as alteration of posterior wing hairs was associated with expression of the PKN kinase domain in the wing margin (Betson and Settleman 2007). We note that posterior wing margin defects are not seen in *delorean* mutants and this may reflect an anterior wing-specific expression pattern of the *pkn^dln^* allele. Nonetheless, the correspondence of the anterior wing margin phenotypes demonstrates that misexpression of the *delorean*-specific transcript behaves similarly to the overexpression of the PKN kinase domain lacking the normal amino-terminal regulatory domains that mediate Rho-GTPase binding (Betson and Settleman 2007). The N-terminal region of PKN is autoinhibitory and it is thought to restrict the kinase activity of the catalytic domain in the absence of activators such as Rho1 (Yoshinaga *et al.* 1999). If the kinase activity of ectopically expressed *pkn* can be activated (by Rho1 or another putative activator Rac1), it would be equivalent to overexpression of the kinase domain. Thus misexpression of the *pkn* transcript, like overexpression of the kinase domain of PKN, can cause alterations in anterior wing margin morphogenesis.

### Involvement of *pkn* in wing morphogenesis

Genetic interactions between *pkn* and the *bsk* and *LIMK1* genes have previously been examined in the context of the loss-of-function allele *pkn^06736^* (Lu and Settleman 1999; Chen *et al.* 2004). Our examination reveals that the essential nature of these interactions is maintained in the context of the *pkn^dln^* allele and can be expanded to include the process of wing morphogenesis. We observed that reduction of *Lim Kinase 1* has no significant effect on the *delorean* wing phenotype. Lack of a notable interaction between *pkn^06736^* and overexpression of LimK1 was previously observed and found to be in contrast to the considerable impact that loss of Rho1 had on suppression of the wing phenotype associated with overexpression of LimK1 (Chen *et al.* 2004). This finding is consistent with the notion that PKN is just one of multiple target effectors that function downstream of Rho1 in any given process or tissue, especially one that would involve a diverse array of responses as needed during wing morphogenesis.

Lu and Settleman (1999) found that reduced levels of a component of the JNK signaling pathway (*bsk*; D-JNK) causes an increase in the dorsal closure defects associated with loss of PKN function in germline clones. We see a similar enhancement of the *delorean* wing phenotype when the dose of *bsk* is reduced. This extends the observation that the function of the Rho1-PKN and JNK signaling cascades “converge at some point” in their function as regulators during embryogenesis (Lu and Settleman 1999) to processes occurring during wing morphogenesis. The enhancement of the *delorean* phenotype upon reduction of Nmo, the founding member of the Nemo-like kinase family of kinases, adds additional evidence in this regard. Nemo-like kinases coordinate the activity of multiple signals as they mediate cross talk between various pathways (Ishitani and Ishitani 2013). In such a capacity, *Drosophila* Nmo plays a key regulatory role as an antagonist of the Wingless signaling pathway during patterning of the developing wing (Zeng and Verheyen 2004). It has also been implicated in the regulation of the JNK pathway and programmed cell death (Mirkovic *et al.* 2002) probably by a process of “morphogenetic apoptosis,” whereby JNK-mediated apoptosis is activated to correct developmental patterning errors (Igaki 2009). Thus, simultaneous reduction of Nmo and misexpression of PKN could have disruptive effects on the integration of a number of signaling pathways involved in wing morphogenesis—Wingless, Rho1, JNK. The observed enhancement of the *delorean* phenotype upon reduction of a regulator (Nmo) and component (D-JNK) of the JNK pathway leads us to speculate about the possibility that PKN may be involved in mediating the previously established dynamic relationship between Rho1 and activation of apoptosis via the JNK pathway (Neisch *et al.* 2010). We note that in this role PKN might not be the only downstream effector target of Rho1 given the observation that dorsal closure defects in Rho1 mutants cannot be rescued by elevated levels of PKN (Lu and Settleman 1999). Nonetheless, our results add weight to the argument that PKN acts as an effector of Rho1 signaling and expands its possible function to wing morphogenesis.

### The role of Pkn as a Rho1 effector

Rho GTPases are critical participants in the orchestration of modifications to cell architecture that must occur during tissue morphogenesis. The array of different biological processes that can be regulated by Rho GTPases coupled with the significant degree of coordination that occurs between them makes an analysis of Rho GTPase signaling pathways a challenge. One approach to understanding the function of Rho GTPases is to determine key effectors in a given signaling event, in a specific tissue. We report here on the development of such a system in *Drosophila* that will allow us to more fully examine the role of the Rho1-Pkn pathway in wing morphogenesis.

## Supplementary Material

Supporting Information
