# Supplementary material for: Disruption of the protein kinase N gene of Drosophila melanogaster Results in the Recessive delorean Allele (pkndln) With a Negative Impact on Wing Morphogenesis
Source: G3 (Bethesda). 2014 Feb 13;4(4):643–56. doi: 10.1534/g3.114.010579 (PMC4059237; doi:10.1534/g3.114.010579)
Supplement: Supporting Information [file supp_4_4_643__index.html]

Disruption of the protein kinase N gene of Drosophila melanogaster Results in the Recessive delorean Allele (pkndln) With a Negative Impact on Wing Morphogenesis — Supporting Information 

# Disruption of the *protein kinase N* gene of *Drosophila melanogaster* Results in the Recessive *delorean* Allele (*pkndln*) With a Negative Impact on Wing Morphogenesis

## Supporting Information for Sass and Ostrow, 2014

**Files in this Data Supplement:**

- Supporting Information - Figures S1-S2 and Tables S1-S2 (PDF, 294 KB)
- Figure S1 - Supplemental characterization of *delorean* wing morphology and wild-type controls. (PDF, 203 KB)
- Figure S2 - Wing morphology of flies expressing double-stranded RNA of *protein kinase N* driven by various GAL4 wing drivers. (PDF, 173 KB)
- Table S1 - Bloomington stocks used for RNA interference experiments. (PDF, 136 KB)
- Table S2 - Quantitative analysis of wing sensory structures in various genetic combinations of GAL4 wing drivers and *pkn*RNAi. (PDF, 161 KB)
