# Supplementary material for: Disruption of the protein kinase N gene of Drosophila melanogaster Results in the Recessive delorean Allele (pkndln) With a Negative Impact on Wing Morphogenesis
Source: G3 (Bethesda). 2014 Feb 13;4(4):643–56. doi: 10.1534/g3.114.010579 (PMC4059237; doi:10.1534/g3.114.010579)
Supplement: Supporting Information [file supp_g3.114.010579_010579SI.pdf]

G3: Genes | Genomes | Genetics

Disruption of the protein kinase N gene of Drosophila melanogaster results in the recessive delorean allele (pkn^{dln}) with a negative impact on wing morphogenesis

Georgette L. Sass and Bruce D. Ostrow

Department of Biology, Grand Valley State University, Allendale, MI 49401

Corresponding author: Dr. Georgette L. Sass, Grand Valley State University, Department of Biology, 1 Campus Drive, Allendale, MI 49401. Email: sassg@gvsu.edu

DOI: 10.1534/g3.114.010579

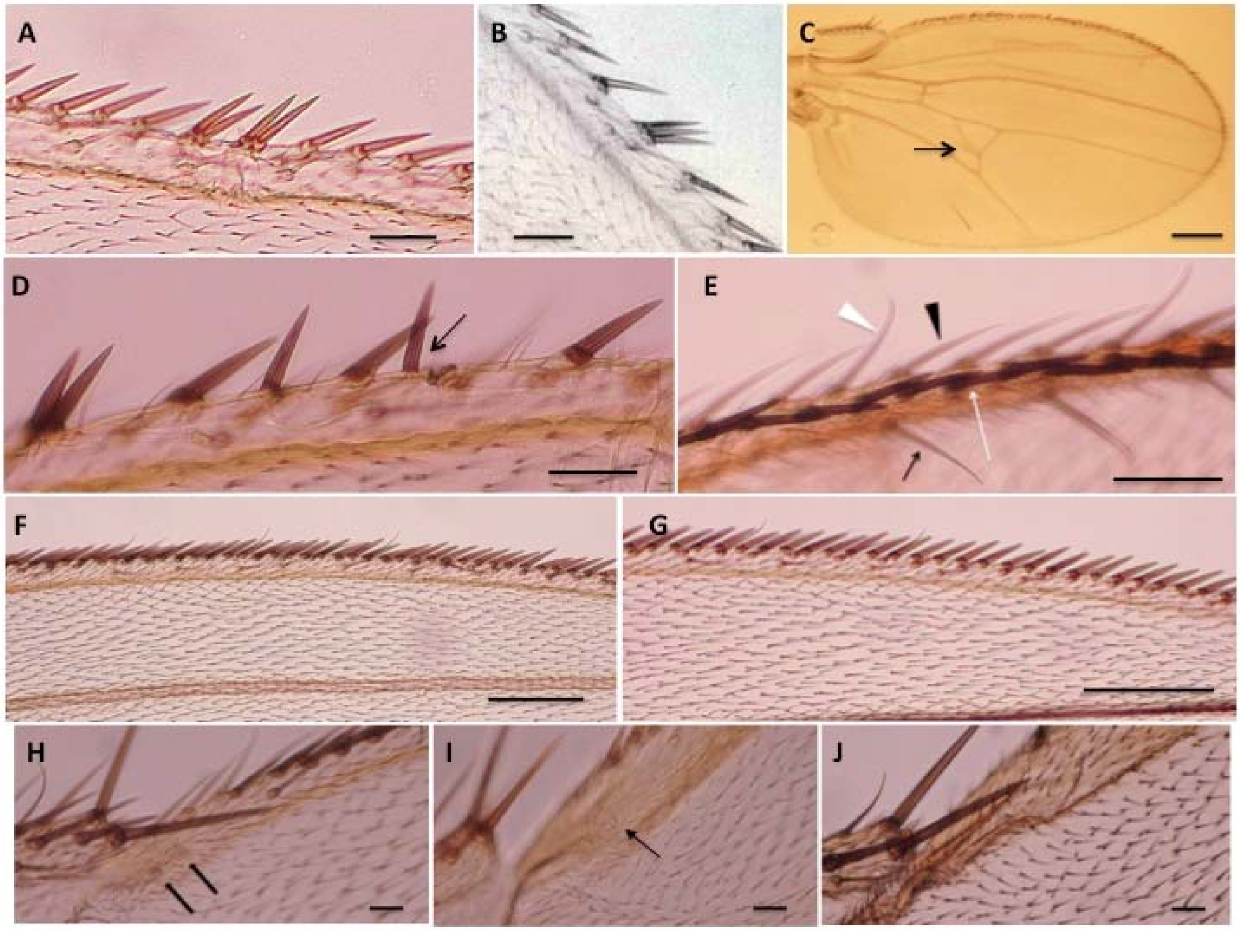

Figure S1 Supplemental characterization of *delorean* wing morphology and wild-type controls. (A) Anterior wing margin of a yw^{1118}; pkn^{dln}/pkn^{dln} homozygote showing one pair of stout bristles that share a socket and one pair of stout bristles that do not share a socket but are spaced very closely. Scale bar = 30µm. (B) Anterior wing margin of a yw^{1118}; pkn^{dln}/pkn^{dln} homozygote showing a triple-stout bristle cluster. Bar = 30µm. C) Dissected wing of w^{1118}; pkn^{dln}/pkn^{dln} homozygote showing ectopic posterior crossvein material (arrow). Bar = 0.2mm. (D) Anterior wing margin of a w^{1118}; pkn^{dln}/pkn^{dln} homozygote showing a stout bristle lacking a socket (arrow). Bar = 30µm. (E) The wild-type anterior wing margin possesses a dorsal row of recurved chemosensory bristles (black arrow), medial row of stout mechanosensory bristles (white arrow), and ventral row of recurved chemosensory bristles (white arrowhead) and slender mechanosensory bristles (black arrowhead). Bar = 50µm. (F) Anterior wing margin of a pkn^{dln}/pkn^{dln}; *Dp(2;3)eve*$^{1.18}$/+ fly that exhibits the wild-type phenotype. Bar = 0.1mm. (G) Anterior wing margin of a pkn^{dln}/*Df(2)w45-30n* fly that exhibits the wild-type phenotype. Bar = 0.1mm. (H) Wild-type wings have two twin campaniform sensillae (arrows) at the proximal end of longitudinal vein L1. Bar = 20µm. (I) An example of a yw^{1118}; pkn^{dln}/pkn^{dln} homozygote wing with only one campaniform sensillum (arrow). Bar = 20µm. (J) An example of a w^{1118}; pkn^{dln}/pkn^{dln} homozygote wing lacking campaniform sensillae. Bar = 20µm.

Table S1 Bloomington stocks used for RNA interference experiments.

Stock number	Genetic component	Expression pattern
28335	*UAS-pknRNAi*	Double-stranded RNA for RNAi of *Pkn* under UAS control
5138	α*Tubulin-GAL4*	GAL4 ubiquitously
3954	*Actin5C-GAL4*	GAL4 ubiquitously
1774	*P{GawB}*69B	GAL4 generally in ectoderm including the wing disc
32544	*P{GawB}c409*	GAL4 in the anterior wing disc
3041	*apterous-GAL4*	GAL4 in an *apterous*$^{+}$ pattern
27327	*cut-GAL4*	GAL4 in the anterior wing margin
1553	*dpp-GAL4*	GAL4 in *decapentaplegic*$^{+}$ pattern
8229	*vestigial-GAL4*	GAL4 in wing blade, wing margin, and wing veins

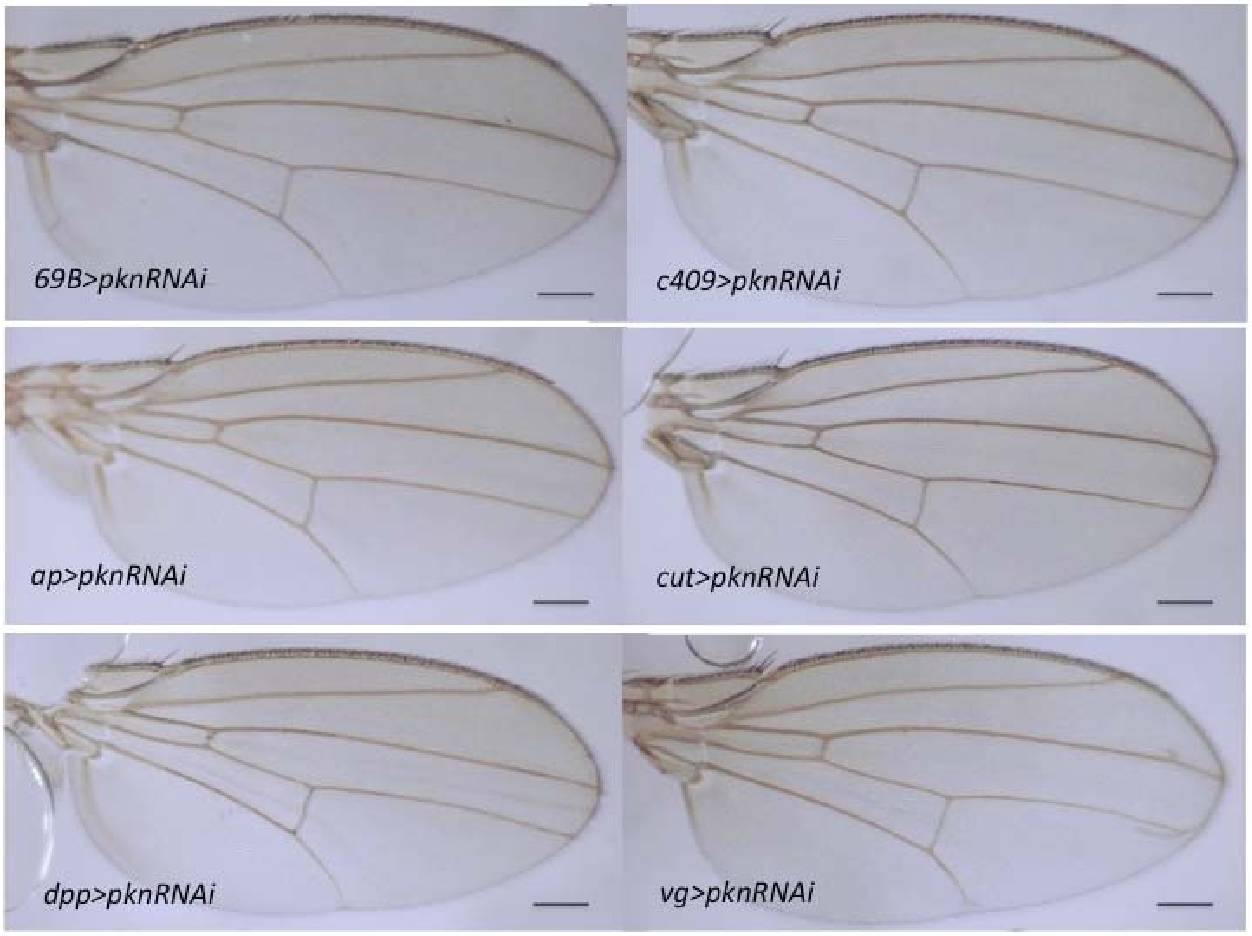

Figure S2 Wing morphology of flies expressing double-stranded RNA of *protein kinase N* driven by various GAL4 wing drivers. Scale bar = 0.2mm. Virgin flies carrying the UAS-*pkn*RNAi transgene were crossed separately to males carrying various GAL4 drivers. Wings of offspring were examined for morphological defects. In almost all genetic combinations, the wings show a wild-type phenotype. In the wing where *vg-GAL4* is driving *pkn*RNAi (lower right panel), the ectopic vein material at the distal end of longitudinal vein L4 is also seen in the *vg-GAL4* strain alone. Only wings of females are shown; male wings are similarly wild-type.

Table S2 Quantitative analysis of wing sensory structures in various genetic combinations of GAL4 wing drivers and *pkn*RNAi. Average number is given along with standard deviation and number of wings scored (sample size). Top rows are values for females; bottom rows (shaded) are values for males. nd = not determined.

	Genotype					
	69B>pknRNAi	*c409>pknRNAi*	*ap>pknRNAi*	*cut>pknRNAi*	*dpp>pknRNAi*	*vg>pknRNAi*
Twin Sensillae	2 ± 0 (11)	2 ± 0 (4)	2 ± 0 (12)	2 ± 0 (5)	2 ± 0 (11)	2 ± 0 (4)
	2 ± 0 (8)	2 ± 0 (5)	nd	2 ± 0 (22)	2 ± 0 (10)	2 ± 0 (5)
Stout	83.14 ± 3.94 (14)	87.83 ± 3.49 (6)	75.21 ± 3.36 (11)	84.33 ± 2.25 (6)	85.73 ± 3.13 (11)	87.4 ± 1.14 (5)
	77.31 ± 2.66 (13)	80.8 ± 2.59 (5)	nd	76.28 ± 2.56 (25)	76.82 ± 3.8 (11)	79.83 ± 3.71 (6)
Ventral Recurved	18.58 ± 1.31 (12)	18.83 ± 0.98 (6)	16.86 ± 1.15 (21)	17.5 ± 1.38 (6)	17.8 ± 1.32 (10)	17.4 ± 0.55 (5)
	17.9 ± 1.37 (10)	18.8 ± 0.84 (5)	nd	17.65 ± 1.19 (23)	17.0 ± 1.12 (9)	17.5 ± 0.84 (6)
