# Supplementary material for: Disruption of the protein kinase N gene of Drosophila melanogaster Results in the Recessive delorean Allele (pkndln) With a Negative Impact on Wing Morphogenesis
Source: G3 (Bethesda). 2014 Feb 13;4(4):643–56. doi: 10.1534/g3.114.010579 (PMC4059237; doi:10.1534/g3.114.010579)
Supplement: Supporting Information [file supp_g3.114.010579_FigureS1.pdf]

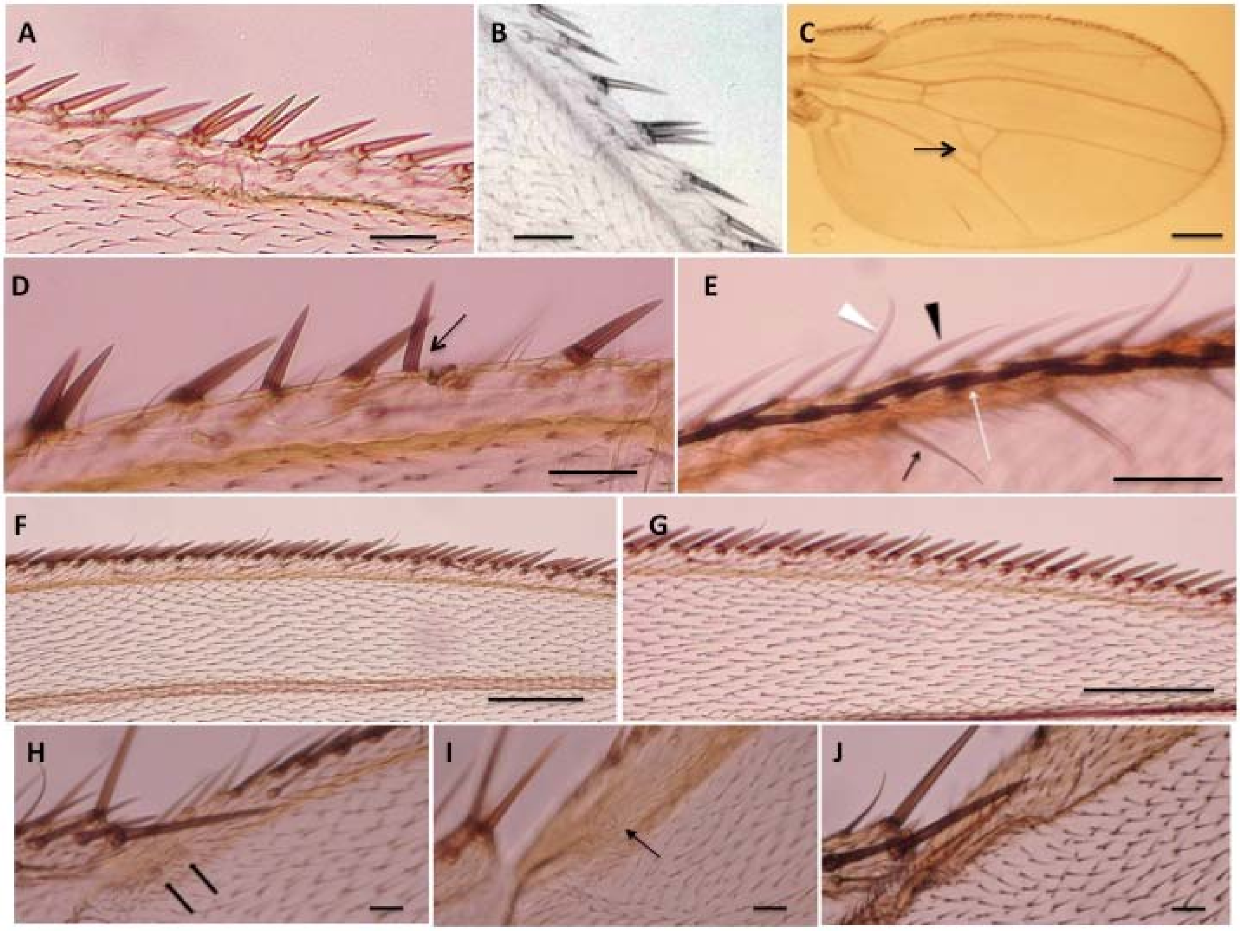

Figure S1 Supplemental characterization of *delorean* wing morphology and wild-type controls. (A) Anterior wing margin of a *yw*^{1118}*; pkn*^{dln}*/ pkn*^{dln} homozygote showing one pair of stout bristles that share a socket and one pair of stout bristles that do not share a socket but are spaced very closely. Scale bar = 30µm. (B) Anterior wing margin of a *yw*^{1118}*; pkn*^{dln}*/ pkn*^{dln} homozygote showing a triple-stout bristle cluster. Bar = 30µm. C) Dissected wing of *w*^{1118}*; pkn*^{dln}*/ pkn*^{dln} homozygote showing ectopic posterior crossvein material (arrow). Bar = 0.2mm. (D) Anterior wing margin of a *w*^{1118}*; pkn*^{dln}*/ pkn*^{dln} homozygote showing a stout bristle lacking a socket (arrow). Bar = 30µm. (E) The wild-type anterior wing margin possesses a dorsal row of recurved chemosensory bristles (black arrow), medial row of stout mechanosensory bristles (white arrow), and ventral row of recurved chemosensory bristles (white arrowhead) and slender mechanosensory bristles (black arrowhead). Bar = 50µm. (F) Anterior wing margin of a *pkn*^{dln}*/pkn*^{dln}; *Dp(2;3)eve*$^{1.18}$/+ fly that exhibits the wild-type phenotype. Bar = 0.1mm. (G) Anterior wing margin of a *pkn*^{dln}*/Df(2)w45-30n* fly that exhibits the wild-type phenotype. Bar = 0.1mm. (H) Wild-type wings have two twin campaniform sensillae (arrows) at the proximal end of longitudinal vein L1. Bar = 20µm. (I) An example of a *yw*^{1118}*; pkn*^{dln}*/ pkn*^{dln} homozygote wing with only one campaniform sensillum (arrow). Bar = 20µm. (J) An example of a *w*^{1118}*; pkn*^{dln}*/ pkn*^{dln} homozygote wing lacking campaniform sensillae. Bar = 20µm.
