# Supplementary material for: Disruption of the protein kinase N gene of Drosophila melanogaster Results in the Recessive delorean Allele (pkndln) With a Negative Impact on Wing Morphogenesis
Source: G3 (Bethesda). 2014 Feb 13;4(4):643–56. doi: 10.1534/g3.114.010579 (PMC4059237; doi:10.1534/g3.114.010579)
Supplement: Supporting Information [file supp_g3.114.010579_FigureS2.pdf]

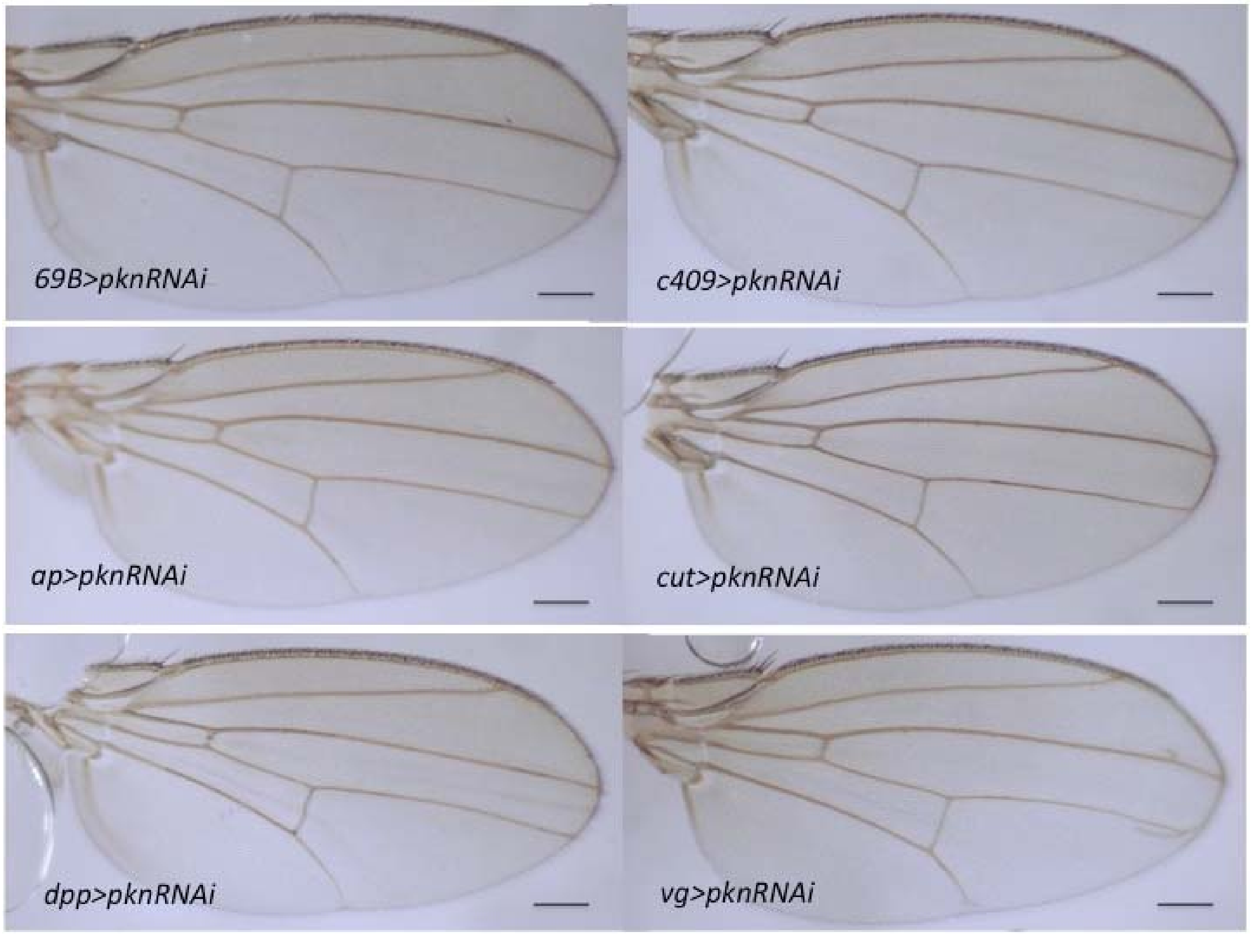


**Figure S2** Wing morphology of flies expressing double-stranded RNA of *protein kinase N* driven by various GAL4 wing drivers. Scale bar = 0.2mm. Virgin flies carrying the UAS-*pkn*RNAi transgene were crossed separately to males carrying various GAL4 drivers. Wings of offspring were examined for morphological defects. In almost all genetic combinations, the wings show a wild-type phenotype. In the wing where *vg-GAL4* is driving *pkn*RNAi (lower right panel), the ectopic vein material at the distal end of longitudinal vein L4 is also seen in the *vg-GAL4* strain alone. Only wings of females are shown; male wings are similarly wild-type.
