# Supplementary material for: Disruption of the protein kinase N gene of Drosophila melanogaster Results in the Recessive delorean Allele (pkndln) With a Negative Impact on Wing Morphogenesis
Source: G3 (Bethesda). 2014 Feb 13;4(4):643–56. doi: 10.1534/g3.114.010579 (PMC4059237; doi:10.1534/g3.114.010579)
Supplement: Supporting Information [file supp_g3.114.010579_TableS1.pdf]

Table S1 Bloomington stocks used for RNA interference experiments.

Stock number	Genetic component	Expression pattern
28335	*UAS-pknRNAi*	Double-stranded RNA for RNAi of *Pkn* under UAS control
5138	α*Tubulin-GAL4*	GAL4 ubiquitously
3954	*Actin5C-GAL4*	GAL4 ubiquitously
1774	*P{GawB}*69B	GAL4 generally in ectoderm including the wing disc
32544	*P{GawB}c409*	GAL4 in the anterior wing disc
3041	*apterous-GAL4*	GAL4 in an *apterous*$^{+}$ pattern
27327	*cut-GAL4*	GAL4 in the anterior wing margin
1553	*dpp-GAL4*	GAL4 in *decapentaplegic*$^{+}$ pattern
8229	*vestigial-GAL4*	GAL4 in wing blade, wing margin, and wing veins
