# Supplementary material for: Disruption of the protein kinase N gene of Drosophila melanogaster Results in the Recessive delorean Allele (pkndln) With a Negative Impact on Wing Morphogenesis
Source: G3 (Bethesda). 2014 Feb 13;4(4):643–56. doi: 10.1534/g3.114.010579 (PMC4059237; doi:10.1534/g3.114.010579)
Supplement: Supporting Information [file supp_g3.114.010579_TableS2.pdf]

Table S2 Quantitative analysis of wing sensory structures in various genetic combinations of GAL4 wing drivers and *pkn*RNAi. Average number is given along with standard deviation and number of wings scored (sample size). Top rows are values for females; bottom rows (shaded) are values for males. nd = not determined.

	Genotype					
	69B>pknRNAi	*c409>pknRNAi*	*ap>pknRNAi*	*cut>pknRNAi*	*dpp>pknRNAi*	*vg>pknRNAi*
Twin Sensillae	2 ± 0 (11)	2 ± 0 (4)	2 ± 0 (12)	2 ± 0 (5)	2 ± 0 (11)	2 ± 0 (4)
	2 ± 0 (8)	2 ± 0 (5)	nd	2 ± 0 (22)	2 ± 0 (10)	2 ± 0 (5)
Stout	83.14 ± 3.94 (14)	87.83 ± 3.49 (6)	75.21 ± 3.36 (11)	84.33 ± 2.25 (6)	85.73 ± 3.13 (11)	87.4 ± 1.14 (5)
	77.31 ± 2.66 (13)	80.8 ± 2.59 (5)	nd	76.28 ± 2.56 (25)	76.82 ± 3.8 (11)	79.83 ± 3.71 (6)
Ventral Recurved	18.58 ± 1.31 (12)	18.83 ± 0.98 (6)	16.86 ± 1.15 (21)	17.5 ± 1.38 (6)	17.8 ± 1.32 (10)	17.4 ± 0.55 (5)
	17.9 ± 1.37 (10)	18.8 ± 0.84 (5)	nd	17.65 ± 1.19 (23)	17.0 ± 1.12 (9)	17.5 ± 0.84 (6)
